# Displaced retinal ganglion cells in albino and pigmented rats

**DOI:** 10.3389/fnana.2014.00099

**Published:** 2014-10-06

**Authors:** Francisco M. Nadal-Nicolás, Manuel Salinas-Navarro, Manuel Jiménez-López, Paloma Sobrado-Calvo, María P. Villegas-Pérez, Manuel Vidal-Sanz, Marta Agudo-Barriuso

**Affiliations:** ^1^Instituto Murciano de Investigación Biosanitaria-Virgen de la ArrixacaMurcia, Spain; ^2^Departamento de Oftalmología, Facultad de Medicina, Universidad de MurciaMurcia, Spain; ^3^Hospital Clínico Universitario Virgen de la ArrixacaMurcia, Spain

**Keywords:** ipsilateral, fluorogold, tracing, Brn3a, axotomy, glaucoma, spatial distribution, intrinsically photosensitive RGCs

## Abstract

We have studied in parallel the population of displaced retinal ganglion cells (dRGCs) and normally placed (orthotopic RGCs, oRGCs) in albino and pigmented rats. Using retrograde tracing from the optic nerve, from both superior colliculi (SC) or from the ipsilateral SC in conjunction with Brn3 and melanopsin immunodetection, we report for the first time their total number and topography as well as the number and distribution of those dRGCs and oRGCs that project ipsi- or contralaterally and/or that express any of the three Brn3 isoforms or melanopsin. The total number of RGCs (oRGCs+dRGCs) is 84,706 ± 1249 in albino and 90,440 ± 2236 in pigmented, out of which 2383 and 2428 are melanopsin positive (m-RGCs), respectively. Regarding dRGCs: i/ albino rats have a significantly lower number of dRGCs than pigmented animals (0.5% of the total number of RGCs vs. 2.5%, respectively), ii/ dRGCs project massively to the contralateral SC, iii/ the percentage of ipsilaterality is higher for dRGCs than for oRGCs, iv/ a higher proportion of ipsilateral dRGCs is observed in albino than pigmented animals, v/ dRGC topography is very specific, they predominate in the equatorial temporal retina, being densest where the oRGCs are densest, vi/ Brn3a detects all dRGCs except half of the ipsilateral ones and those that express melanopsin, vii/ the proportion of dRGCs that express Brn3b or Brn3c is slightly lower than in the oRGC population, viii/ a higher percentage of dRGCs (13% albino, 9% pigmented) than oRGCs (2.6%) express melanopsin, ix/ few m-RGCs (displaced and orthotopic) project to the ipsilateral SC, x/ the topography of m-dRGCs does not resemble the general distribution of dRGCs, xi/ The soma size in m-oRGCs ranges from 10 to 21 μm and in m-dRGCs from 8 to 15 μm, xii/ oRGCs and dRGCs have the same susceptibility to axonal injury and ocular hypertension. Although the role of mammalian dRGCs remains to be determined, our data suggest that they are not misplaced by an ontogenic mistake.

## Introduction

Retinal ganglion cells (RGCs) are the only afferent neurons of the retina. Their axons form the optic nerve and carry the visual information, pre-processed in the retina, to their image forming and non-image forming target territories in the brain.

As a rule, RGCs are located in the innermost nuclear layer of the retina, the ganglion cell layer (orthotopic RGCs, oRGCs). However, there are some RGCs that are displaced and lay in the inner plexiform layer or inner nuclear layer. These RGCs were first described by Dogiel in the bird retina (Dogiel, 1895) and are known as displaced RGCs (dRGCs). Their function, projections and retinal topography have been thoroughly studied in birds (Karten et al., 1977; Fite et al., 1981; Mey and Johann, 2001) and reptiles (Bellintani-Guardia and Ott, 2002). In the pigeon, chick, and chameleon dRGCs project only to the nBOR (nucleus of the basal root, part of accessory optic nuclei), and control the optokinetic nistagmus (Simpson, 1984; Cook and Podugolnikova, 2001). In mammals, however, projections to the AOS are only from oRGCs (Dann and Buhl, 1987; Cook and Podugolnikova, 2001) while dRGCs in mice and rabbits project mainly to the superior colliculi (Dräger and Olsen, 1980, 1981; Vaney et al., 1981; Balkema and Dräger, 1990).

The function of dRGCs in mammals is unknown. It has been suggested that they are misplaced as a result of an ontogenic aberration rather than representing an independent functional class of RGCs (Buhl and Dann, 1988; Doi et al., 1994).

In terms of their distribution, in rabbits and rats dRGCs are observed across the retina, but their occurrence is biased toward the periphery (Robson and Hollander, 1984; Liu and Jen, 1986). In mouse and Chinese hamster they are more abundant in the peripheral and temporal retina (Dräger and Olsen, 1980; Doi et al., 1994) while in the monkey they are densest around the peripapillary region (Bunt and Minckler, 1977).

In mice, melanin pigmentation greatly determines the population of dRGCs, and albino or hypopigmented animals have lower numbers of these neurons (Dräger and Olsen, 1980; Balkema and Dräger, 1990). Pigmentation affects as well the ipsilateral RGC projection in rats and mice (Lund, 1965; Dräger and Olsen, 1980; Lund et al., 1980; Balkema and Dräger, 1990; Nadal-Nicolás et al., 2012), and thus while in albino and pigmented mice the proportion of ipsilateral dRGCs is higher than of ipsilateral oRGCs, in albino animals both, dRGCs and the ipsilateral projection, are greatly reduced (Dräger and Olsen, 1980).

There is a subpopulation of RGCs that is intrinsically photosensitive through the photopigment melanopsin (m-RGCs). m-RGCs mediate non-image forming visual processes, such as circadian clock rhythm regulation and pupillary constriction, (Schmidt et al., 2011) although they may also have a role in image forming vision (Ecker et al., 2010; Brown et al., 2012; Schmidt et al., 2014). There are 5 subtypes of m-RGCs (M1–M5) which are preferentially found in the ganglion cell layer, except a subtype with M1 and M2 characteristics, termed M-d, that in mice is found displaced to the inner nuclear layer (Berson et al., 2010; Qiu and Goz, 2010; Schmidt et al., 2011; Sand et al., 2012; Karnas et al., 2013).

RGCs can be identified by tracing from the superior colliculi or from the optic nerve, (Lund, 1965; Dräger and Olsen, 1980; Linden and Perry, 1983; Sefton et al., 2004; Salinas-Navarro et al., 2009b,c) being the latter a method by which the whole retinofugal population can be labeled. Among the available tracers, fluorogold (FG) is widely used in the visual system. Although FG is not suitable for long term experiments as its signal fades with time (Sellés-Navarro et al., 1996; Gómez-Ramírez et al., 1999), it is an excellent tracer to identify RGCs with an intact active axonal transport, since FG is retrogradely and actively transported from the axons to the soma, where it accumulates without leaking (reviewed in Köbbert et al., 2000).

There are also specific RGC markers that allow their identification by *ex vivo* methods (Surgucheva et al., 2008; Nadal-Nicolás et al., 2009; Galindo-Romero et al., 2011; Nguyen et al., 2011; Rodriguez et al., 2014). Among these RGC-specific proteins are the neuronal differentiation and survival Pou4f family of transcription factors (Brn3a, Brn3b, and Brn3c) (Badea et al., 2009; Badea and Nathans, 2011). In fact, identification of RGCs by Brn3a immunodetection is a powerful tool to assess RGC survival in several mouse and rat injury models such as ocular hypertension (Salinas-Navarro et al., 2009a, 2010; Cuenca et al., 2010; Vidal-Sanz et al., 2012), traumatic optic nerve injury (Nadal-Nicolás et al., 2009; Galindo-Romero et al., 2011), excitotoxicity (Ganesh and Chintala, 2011; DeParis et al., 2012), optic neuritis (Smith et al., 2011) and retinal degeneration (García-Ayuso et al., 2010), and to quantify the efficacy of neuroprotective therapies (Sánchez-Migallón et al., 2011; Galindo-Romero et al., 2013b).

Previous reports have shown the morphological diversity of albino rat dRGCs and the effect of the enucleation of one eye in this RGC population (Liu and Jen, 1986; Buhl and Dann, 1988). The purpose of this work is to further characterize the dRGC population. Specifically, we have addressed in albino and pigmented rats: i/ their total number and retinal topography, ii/ their projection to one or both superior colliculi (ipsilaterality and contralaterality), iii/, whether they express any of the Brn3 members and/or melanopsin, and, iv/ their response to injury.

## Materials and methods

### Animal handling, anesthesia, and analgesia

Three months old female albino Sprague Dawley (SD, 180–220 g body weight) and pigmented Pievald Virol Glaxo (PVG, 220–250 g body weight) rats were obtained from the University of Murcia breeding colony. All experimental procedures were carried out in accordance with the Association for Research in Vision and Ophthalmology and European Union guidelines for the use of animals in research and were approved by the Ethical and Animal Studies Committee of the University of Murcia (Spain).

#### Animals subjected to surgery

For anesthesia a mixture of xylazine (10 mg/kg body weight; Rompun; Bayer, Kiel, Germany) and ketamine (60 mg/kg body weight; Ketolar®; Pfizer, Alcobendas, Madrid, Spain) was used intraperitoneally (i.p.). After surgery, an ointment containing tobramycin (Tobrex; Alcon S. A., Barcelona, Spain) was applied on the cornea to prevent its desiccation. Rats were given oral analgesia (Buprex, Buprenorphine 0.3 mg/mL, Schering-Plough, Madrid, Spain) at 0.5 mg/kg (prepared in strawberry-flavored gelatine) the day of the surgery and during the next 3 days.

All animals were sacrificed with an i.p. injection of an overdose of pentobarbital (Dolethal, Vetoquinol, Especialidades Veterinarias, S. A., Alcobendas, Madrid, Spain).

### Surgery

#### Tracing the whole dRGC/oRGC population

The supraorbital skin was incised, the superior rectus muscle sectioned and the intraorbital optic nerve exposed. The dural sheath surrounding the optic nerve was opened longitudinally at ~1 mm from the optic disk, and a pledge of gelatine sponge soaked in fluorogold (FG, Fluorochrome, LLC, USA) at 6% diluted in 10% dimethyl-sulfoxide (DMSO) in saline was applied surrounding the nerve. The eye fundus was inspected after the procedure and the animals were processed 3 days later. Because this approach does not imply severing the optic nerve or the blood supply to the retina, both retinas of each animal were traced.

#### Tracing dRGCs/oRGCs projecting to the superior colliculi

A pledge of gelatine sponge soaked in 3%FG, 10% DMSO in saline was applied to both superior colliculi (SC) 1 week prior to animal processing following standard techniques in our laboratory (Lindqvist et al., 2004; Jehle et al., 2008; Nadal-Nicolás et al., 2009; Salinas-Navarro et al., 2009c).

#### Tracing dRGCs/oRGCs projecting ipsilaterally

The left superior colliculus was removed by aspiration (Nadal-Nicolás et al., 2012), this removal ensures that only RGC projections to the right SC will uptake the tracer. One week later, Fluorogold (3%) was applied to the right SC and animals were processed a further week later. Ipsilatetrrally projecting RGCs were studied in the right retinas.

#### Optic nerve transection (ONT)

In albino SD rats, the left optic nerve (ON) was intraorbitally transected according to standard procedures in our laboratory (Villegas-Pérez et al., 1993; Vidal-Sanz et al., 2002; Agudo et al., 2009; Nadal-Nicolás et al., 2009; Parrilla-Reverter et al., 2009a,b; Galindo-Romero et al., 2011). The ON was sectioned 0.5 mm from the optic disc sparing the blood supply. After the injury, the eye fundus was checked to verify that the retinal blood supply was intact. Animals were sacrificed 7 days later.

#### Ocular hypertension (OHT)

In albino SD rats the episcleral and perilimbar vessels were cauterized by laser diode using previously reported methods (Salinas-Navarro et al., 2010; Vidal-Sanz et al., 2012; Agudo-Barriuso et al., 2013). Intraocular pressure was monitored before and after the procedure using a rebound tonometer (Tonolab, Tiolat, OY, Helsinki, Finland). The mean ± standard deviation of the intraocular pressure before the surgery was 10.26 ± 0.46 mmHg, and rose to 41.7 ± 10.4 and 31.9 ± 15.5 mmHg at 24 h and 7 days after the procedure, respectively. Animals were sacrificed 14 days after the induction of OHT.

The experimental design and the number of analyzed retinas is detailed in Table 1.

**Table 1:** **Experimental design**.

**Study**	**Animal groups**	**RGC injury**	**Immunodetection**				
			**Melanopsin**	Brn3a^*^	**Brn3b**	**Brn3c**	**Brn3**^#^
dRGCs oRGCs in control retinas	Tracing from the optic nerve: The whole retinofugal population	None	–	SD (7) PVG (6)	–	–	–
	Tracing from both SC: RGCs projecting to the SC	None	–	SD (6) PVG (6)	–	–	–
	Tracing from one SC: Ipsilateral RGCs	None	SD (5) PVG (3)^‡^		SD (4) PVG (3)	SD (5) PVG (3)	
	No tracing: Brn3 or melanopsin	None	SD (6) PVG (6)^‡^		SD (8)^§^ PVG (6)^§^		SD (8) PVG (6)
dRGCs oRGCs response to injury	Tracing from both SC^†^	OHT (14d)	–	SD (20)	–	–	–
	No tracing	ONT (7d)	–	SD (8)	–	–	–

*In brackets is shown the number of retinas analyzed per group and strain (SD: albino, PVG: pigmented). Within each group the whole population of oRGCs and dRGCs either traced and/or immunodetected was analyzed. To quantify the ipsilateral population only FG^+^dRGCs/oRGCs traced from the ipsilateral colliculus were taken into account (14 albino and 9 pigmented retinas). Out of these, some retinas were used to quantify the ipsilateral dRGCs positive for each Brn3 or melanopsin*.

* *Brn3a^+^ oRGCs and dRGCs were counted in 19 albino and in 18 pigmented retinas (sum of retinas traced from the SC, from the ON, and non-traced retinas double immunodetected with melanopsin)*.

§ *Double immunodetection of Brn3b and Brn3c*.

‡ *Double immunodetection of melanopsin and Brn3a*.

# *Triple immunodetection, where the 3 Brn3 primary antibodies were visualized using the same fluorophore. This group served to quantify Brn3^+^ oRGCs and dRGCs*.

† *After ocular hypertension, the impairment of RGC retrograde axonal transport was assessed by FG-tracing from the SC (1 week before processing), while RGC survival was quantified by Brn3a immunodetection*.

### Retinal dissection

Unless otherwise stated, all the reagents were from Sigma-Aldrich, (Alcobendas, Madrid, Spain).

Animals were perfused transcardially with 4% paraformaldehyde (PFA) in phosphate buffer 0.1 M after a saline rinse.

#### Flat mounts

Right after deep anesthesia and before fixation a suture was placed on the dorsal pole of each eye for orientation purposes. Retinas were dissected as flattened whole-mounts by making four radial cuts (the deepest one in the dorsal pole), post-fixed for an additional hour in 4% PFA and kept in phosphate buffered saline (PBS) till further processing as reported (Villegas-Pérez et al., 1996; Sobrado-Calvo et al., 2007; Nadal-Nicolás et al., 2009; Salinas-Navarro et al., 2009b; Ortín-Martínez et al., 2010; Sánchez-Migallón et al., 2011).

#### Cross-sections

Two retinas from albino rats traced with FG applied onto both SC were used. The cornea and crystalline lens were removed and the optic cups were cryoprotected in increasing concentrations of sucrose before embedding them in optimal cutting temperature (OCT) compound (Sakura Finetek, Torrance, CA) for cryostat sectioning (15 μm). Sections were washed in PBS to eliminate the OCT, mounted in antifading medium (Vectashield Mounting Medium, Vector, Atom, Alicante, Spain) and photographed under an epifluorescence microscope (see below).

### Immunohistofluorescence protocol

Immunodetection of flat mounted retinas was carried out as previously described (Nadal-Nicolás et al., 2009, 2012; Galindo-Romero et al., 2011, 2013a; Sánchez-Migallón et al., 2011). FG-traced retinas were always immunodetected for Brn3a. Brn3b and Brn3c were single or double immunodetected. In some retinas, Brn3a, Brn3b and Brn3b were triple immunodetected and developed using the same fluorophore to assess the whole population of Brn3-expressing oRGCs/dRGCs. Melanopsin was always double immunodetected with Brn3a (Table 1).

### Antibodies and working dilutions

#### Primary antibodies

Goat anti-Brn3a (C-20) (dilution 1:750), goat anti-Brn3b (H-18) (dilution 1:50), mouse anti-Brn3c (QQ8) (dilution 1:250) all from Santa Cruz Biotechnologies (Heidelberg, Germany), and rabbit anti-melanopsin (PAI-780) (dilution 1:1000) from Thermo Scientific (Madrid, Spain).

#### Secondary antibodies

Donkey anti-goat Dylight 594 (Jackson Immuno-Research, Newmarket, Suffolk, UK); donkey anti-goat Alexa 488, donkey anti-mouse Alexa 594, donkey anti-rabbit Alexa 594 all from Molecular Probes (Life Technologies, Madrid, Spain). All secondary antibodies were used at 1:500 dilution.

### Antibody characterization

The mouse monoclonal IgG_1_ against Brn3c is raised using as immunogen the human recombinant Brn3c. In the rodent retina specifically detects a subset of RGCs (Jain et al., 2012; Nadal-Nicolás et al., 2012).

The goat polyclonal IgG Brn3b is raised against an internal region of human Brn3b and recognizes a single band of 55 kD on Western blots from mouse eye extracts according to the manufacturer. In the mouse and rat retina specifically labels a subset of RGCs (Nie et al., 2010; Nadal-Nicolás et al., 2012).

The goat polyclonal IgG to Brn3a is raised against the N-terminus of human Brn3a. This antibody specifically immunolabels the vast majority of rat and mouse RGCs (Nadal-Nicolás et al., 2009; Galindo-Romero et al., 2011) and has been extensively used to identify and quantify RGCs in these species (Burugula et al., 2011; Smith et al., 2011; Nadal-Nicolás et al., 2012; Vidal-Sanz et al., 2012; Galindo-Romero et al., 2013a,b).

The rabbit polyclonal to melanopsin is raised using a synthetic peptide corresponding to the N-terminal 1–19 residues of rat melanopsin. This antibody is the rat version of Provencio's UF006 anti-mouse melanopsin antibody (Provencio et al., 2002) and recognizes both, short and long, melanopsin isoforms (Hughes et al., 2012; Nadal-Nicolás et al., 2012; Galindo-Romero et al., 2013a).

### Image acquisition

All retinas were photographed with an epifluorescence microscope (Axioscop 2 Plus; Zeiss Mikroskopie, Jena, Germany) equipped with a computer-driven motorized stage (ProScan™ H128 Series, Prior Scientific Instruments, Cambridge, UK), controlled by the Image Pro Plus software, (IPP 5.1 for Windows®; Media Cybernetics, Silver Spring, MD, USA), as previously described (Nadal-Nicolás et al., 2009, 2012; Salinas-Navarro et al., 2009b; Galindo-Romero et al., 2013a). Briefly: to make reconstructions of retinal whole-mounts, retinal multi-frame acquisitions were taken for each marker in a raster scan pattern. Frames (154 per retina) were captured contiguously side-by-side with no gap or overlap between them using a x10 objective (Plan-Neofluar, 10x/0.30; Zeiss Mikroskopie, Jena, Germany). Then, the individual frames were combined automatically into a single tiled high resolution photomontage using IPP® for Windows®. Retinal magnifications were acquired with x20 or x40 objectives and their brightness and contrast were adjusted using Adobe Photoshop CS3 v10.0 (Adobe System Incorporated, USA).

### Identification of dRGCs

The whole retinas were photographed focusing in the ganglion cell layer using different fluorescence filters to acquire FG, Brn3 or melanopsin signal and photomontages of each retina were generated. By changing the microscope focus from the GCL to the inner plexiform layer and inner nuclear layer, the position of dRGCs FG, Brn3 or melanopsin positive was manually dotted in the retinal photomontage. The number of dots representing dRGCs was automatically quantified as detailed below.

### Quantification and spatial distribution of oRGCs

FG-traced, melanopsin positive or Brn3 immunodetected RGCs in the ganglion cell layer (oRGCs) were automatically quantified using previously described routines (Salinas-Navarro et al., 2009b; Nadal-Nicolás et al., 2012; Galindo-Romero et al., 2013a). The topographical distribution of FG-traced and Brn3^+^ oRGCs was represented by isodensity maps as reported before (Nadal-Nicolás et al., 2009, 2012; Salinas-Navarro et al., 2009b). The distribution of melanopsin^+^RGCs (orthotopic and displaced) was visualized by neighbor maps (see below).

### Quantification of dRGCs

An automatic routine was developed with the IPP software to quantify the total number of dots in each photomontage: first the user was asked to mark the optic nerve (ON) as a reference point in the retina and to draw the retinal contour to measure its total area. Position coordinates (x,y) of each dot were then automatically obtained. Finally, all data including the spatial coordinates and number of dots were exported to a spreadsheet (Office Excel 2000; Microsoft Corp., Redmond, WA) for spatial analysis (see next section).

### dRGCs and m-RGCs spatial distribution: neighbor maps

Because the total population of dRGCs and of m-RGCs (orthotopic or displaced) is small, their topography was assessed using the *k*-nearest neighbor algorithm with a fixed radius of 0.276 mm, as previously described (Galindo-Romero et al., 2013a). This method plots every cell in its retinal position coloring each one according to the number of its neighbors. The warmer the color of the plot, the more dRGCs/m-RGCs cells are in a given retinal location. In addition, maps representing the distribution of dRGCs from 3 superimposed retinas (normalized maps) are used to analyze the distribution of these cells. This was feasible because the retinas were from animals of similar ages, equally oriented and the maps centered on the optic nerve.

The numerical positional data gathered after the spatial analysis allowed as well, the calculation of the number of dRGCs at a given position from the optic nerve. Because dRGCs were more abundant in the temporal quadrant, for the analysis of the number of dRGCs present in each retinal quadrant, the retina was divided in four quadrants: superior, inferior, temporal and nasal (see **Figure 5**). These data were as well represented in bar graphs (number of cells against distance from the optic nerve) using SigmaPlot (SigmaPlot® 9.0 for Windows®; Systat Software, Inc., Richmond, CA, EEUU).

### Sampling and measurement of melanopsin ^+^RGCs soma diameter

In 4 albino and 4 pigmented retinas, 12 samples of 0.1575 mm^2^ were acquired, three per quadrant. The first sample was taken at 0.875 mm from the optic disc and the other two at 1 mm from each other (Nadal-Nicolás et al., 2009). For each sample two images were acquired, one for m-oRGCs and the other for m-dRGCs. Then, using the IPP software, the soma of each m-RGC was detected and its diameter calculated as the averaged length of diameters measured at two degree intervals and passing through cell body centroid. A total of 333 and 381 m-oRGCs and 117 and 73 m-dRGCs from pigmented and albino retinas were analyzed, respectively.

### Statistical analyses

To compare RGC number values from both strains, we used the SigmaStat® for Windows™ Version 3.11 program; (Systat Software, Inc., Richmond, CA). Differences were considered significant when *p* < 0.05 and tests are detailed in results.

## Results

In all retinas both oRGCs and dRGCs were studied in parallel. This allowed us to correlate both populations in number (Tables 2–**6**) and distribution (**Figures 4, 6–10**). Then, as a rule, a given oRGC isodensity map and a dRGC neighbor map are from the same retina and are labeled with the same letter. Besides, each pair of maps from a right and a left retina is from the same albino or pigmented animal. In addition, dRGC neighbor maps from single retinas are shown on a black background while the normalized distributions, i.e., neighbor maps where data from 3 retinas are represented, are on a blue background. Finally, the percentages of oRGCs or dRGCs expressing Brn3 or melanopsin were calculated using as 100% the number of RGCs traced with FG from the optic nerve. In addition, the proportion of m-oRGCs vs. m-dRGCs was calculated using as 100% the total number of melanopsin ^+^RGCs.

**Table 2:** **Number of oRGCs and dRGCs traced from the optic nerve, from both superior colliculi, and from one superior colliculus. Data are shown as the mean ± standard deviation**.

	**Albino**		**Pigmented**	
	**dRGCs**	**oRGCs**	**dRGCs**	**oRGCs**
Traced from the optic nerve (*n* = 6–7/strain)	422 ± 80	84,284 ± 1182	2293 ± 197	88,147 ± 2286
Total number of RGCs (displaced + orthotopic)	84,706 ± 1249		90,440 ± 2236	
Traced from both superior colliculi (*n* = 6/strain)	375 ± 37	82,412 ± 1568	2454 ± 235	86,020 ± 3582
RGCs projecting to both superior colliculi (displaced + orthotopic)	82,789 ± 1579		88,474 ± 3649	
Traced from one superior colliculi (ipsilateral) (*n* = 9–14/strain)	18 ± 4	2064 ± 264	51 ± 12	3548 ± 497
Ipsilateral population (displaced + orthotopic)	2082 ± 262		3599 ± 496	

### Identification of dRGCs in retinal flat-mounts and expression of Brn3 transcription factors

**Figure 1:**
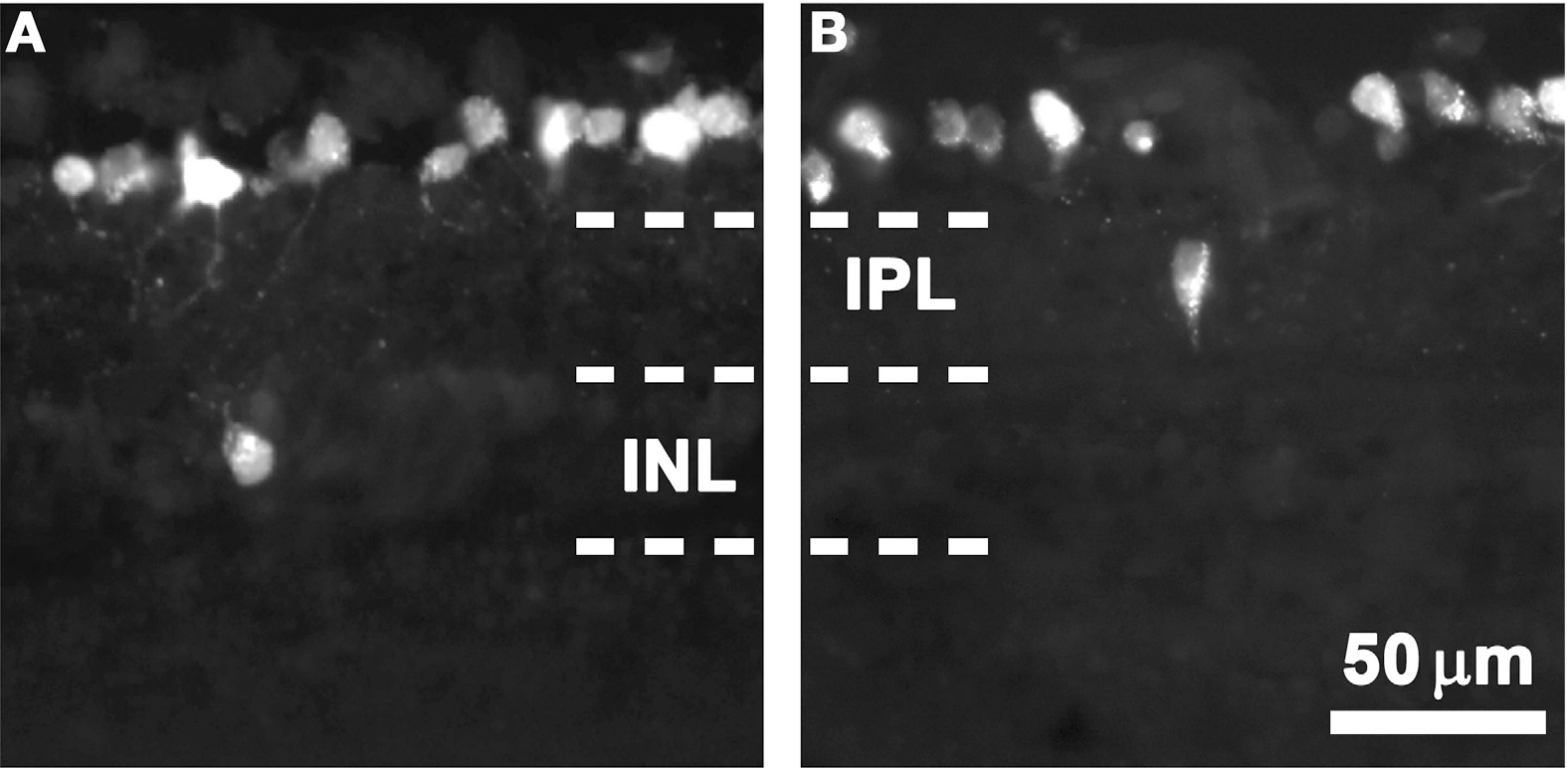
**dRGCs in retinal cross-sections**. Magnifications from albino retinal cross-sections traced with fluorogold applied to both superior colliculi. In these microphotographs is observed that most of the RGCs are located in the ganglion cell layer and that dRGCs are found either in the inner nuclear layer (INL, **A**) or the inner plexiform layer (IPL, **B**). Scale in **(B)**.

As observed in the FG-traced cross-sections shown in Figure 1, most RGCs are placed in the ganglion cell layer (orthotopic, oRGCs), while displaced RGCs (dRGCs) are located either in the inner nuclear layer (Figure 1A) or inner plexiform layer (Figure 1B).

**Figure 2:**
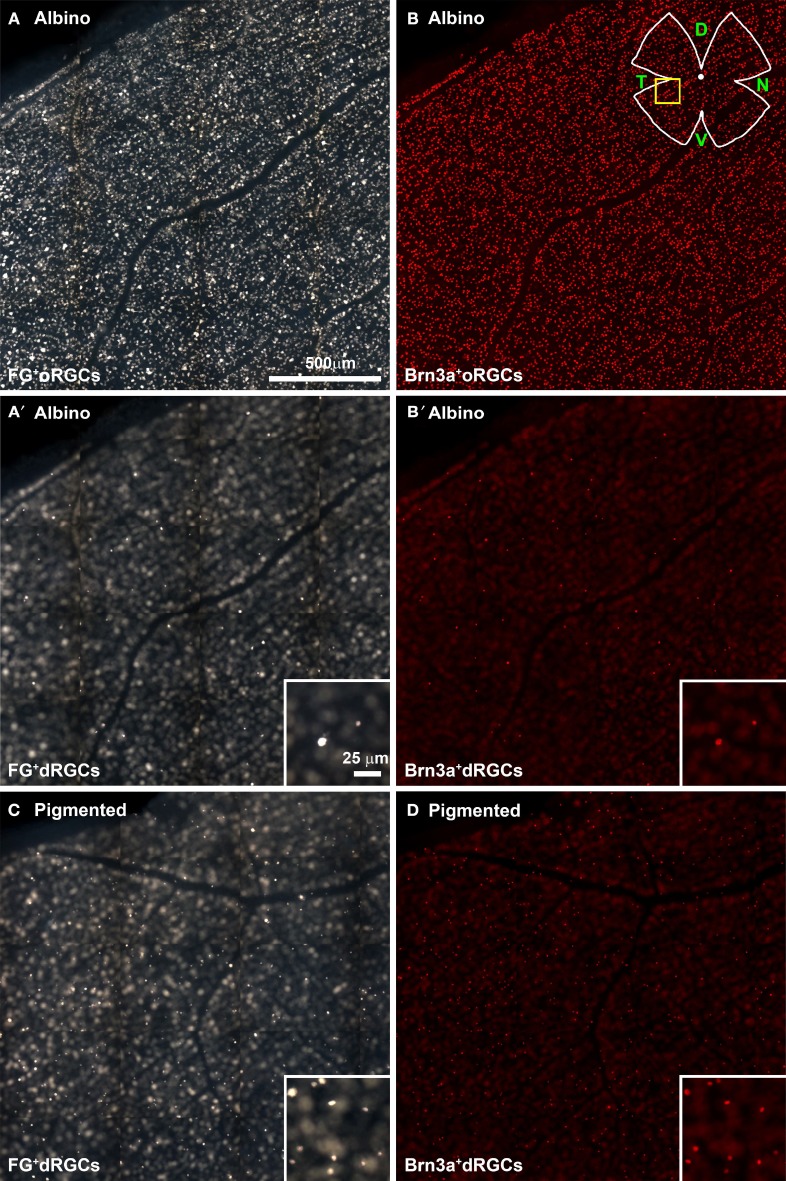
**Identification of fluorogold traced and Brn3a^+^ dRGCs in retinal flat mounts**. Multiframe mosaics from flat-mounted retinas from an albino **(A–B′)** and a pigmented **(C,D)** retina taken with different fluorescent filters and focus to illustrate the technique used to photograph the retina and to observe oRGCs and dRGCs. **(A,B)** Fluorogold-traced **(A)** and Brn3a^+^ **(B)** oRGCs in albino rats. In these same areas but focusing in the inner plexiform layer or inner nuclear layer, FG-traced and Brn3a^+^ dRGCs are identified **(A′,B′)**. In **(C,D)** are shown FG-traced **(C)** and Brn3a^+^ **(D)** dRGCs in the pigmented strain. These mosaics are from the retinal area shown in the yellow square (drawing in **B**) and are tiles of 15 individual frames taken with an x20 objective (a whole photomontage spans 154 frames acquired with a x10 objective). D, dorsal; T, temporal; N, nasal; V, ventral.

In FG-traced and Brn3a immunodetected retinal flat-mounts (Figure 2, Supplementary Figure 1), dRGCs are observed when changing the microscope focus from the ganglion cell layer (Figures 2A,B) to more external retinal layers (Figures 2A′,B′).

In the retinal whole-mounts, it was observed that: (i) most of the dRGCs express Brn3a (compare Figure 2A with Figure 2A′ and Figure 2C with Figure 2D), and (ii) dRGCs are more abundant in the pigmented than in the albino strain (compare Figure 2A′ with 2C and 2B′ with 2D). This is addressed in detail below.

**Figure 3:**
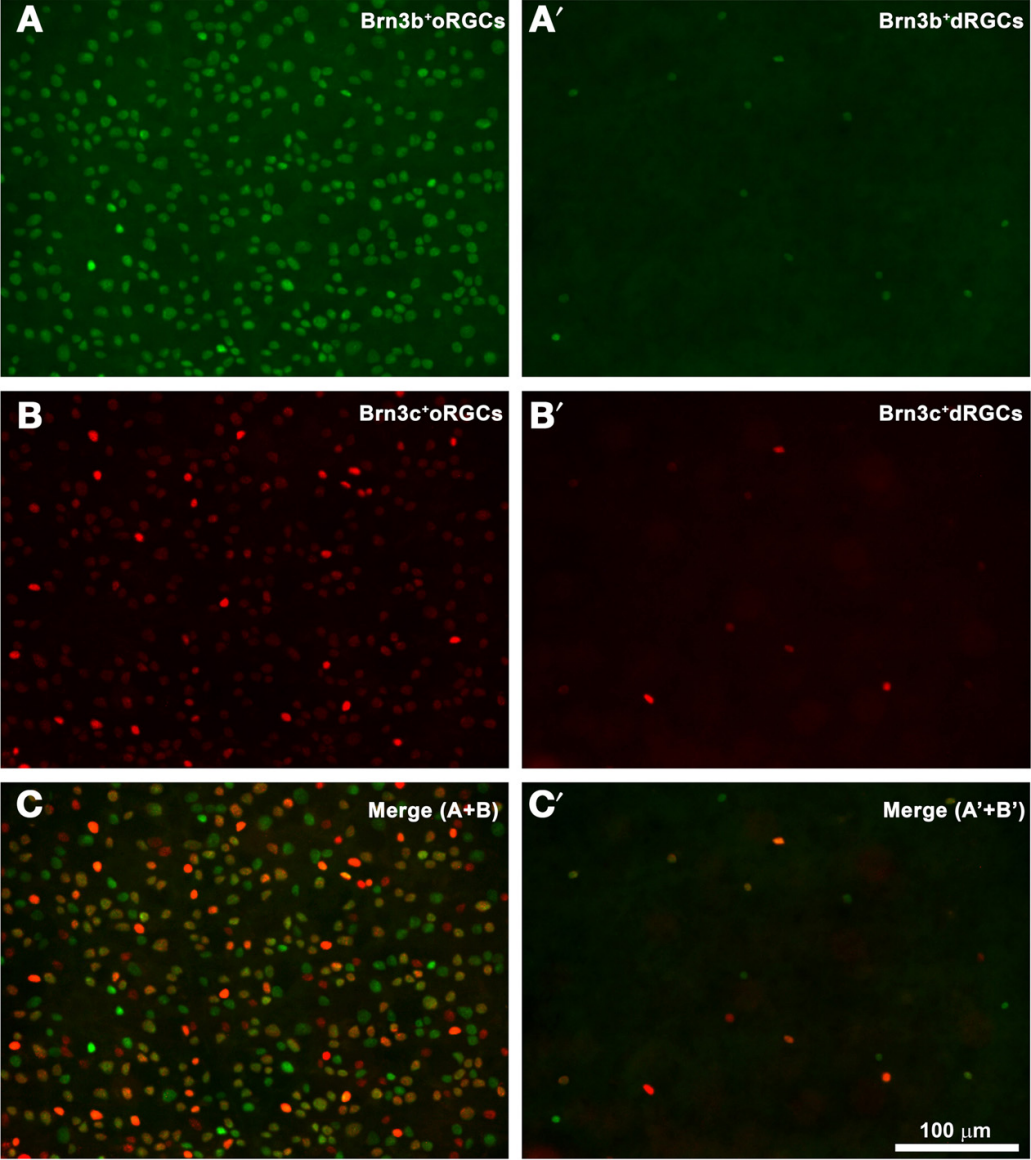
**dRGCs and Brn3b or Brn3c expression. (A,B)** Magnification from a flat mounted retina focused on the ganglion cell layer where Brn3b^+^ **(A)** or Brn3c^+^ **(B)** oRGCs are observed. In this same frame but focusing in the inner plexiform layer, dRGCs expressing Brn3b **(A′)** or Brn3c **(B′)** were photographed. **(C,C′)** are the merged images of Brn3b and Brn3c positive RGCs **(C)** or dRGCs **(C′)**. Bar scale in **(C′)**.

Do dRGCs express as well the other two members of the Brn3 family? Figure 3 shows a magnification of the same area from a pigmented rat retina where Brn3b^+^ and Brn3c^+^ oRGCs (Figures 3A,B, respectively, merged in Figure 3C) and dRGCs (Figures 3A′,B′, respectively, merged in Figure 3C′) are observed. These images were acquired from within the area framed in Figure 2B (drawing) which is the retinal region with higher dRGC densities (see below), and confirm that Brn3b and Brn3c are expressed in a proportion of dRGCs (see details below).

### Total number and distribution of dRGCs

**Figure 4:**
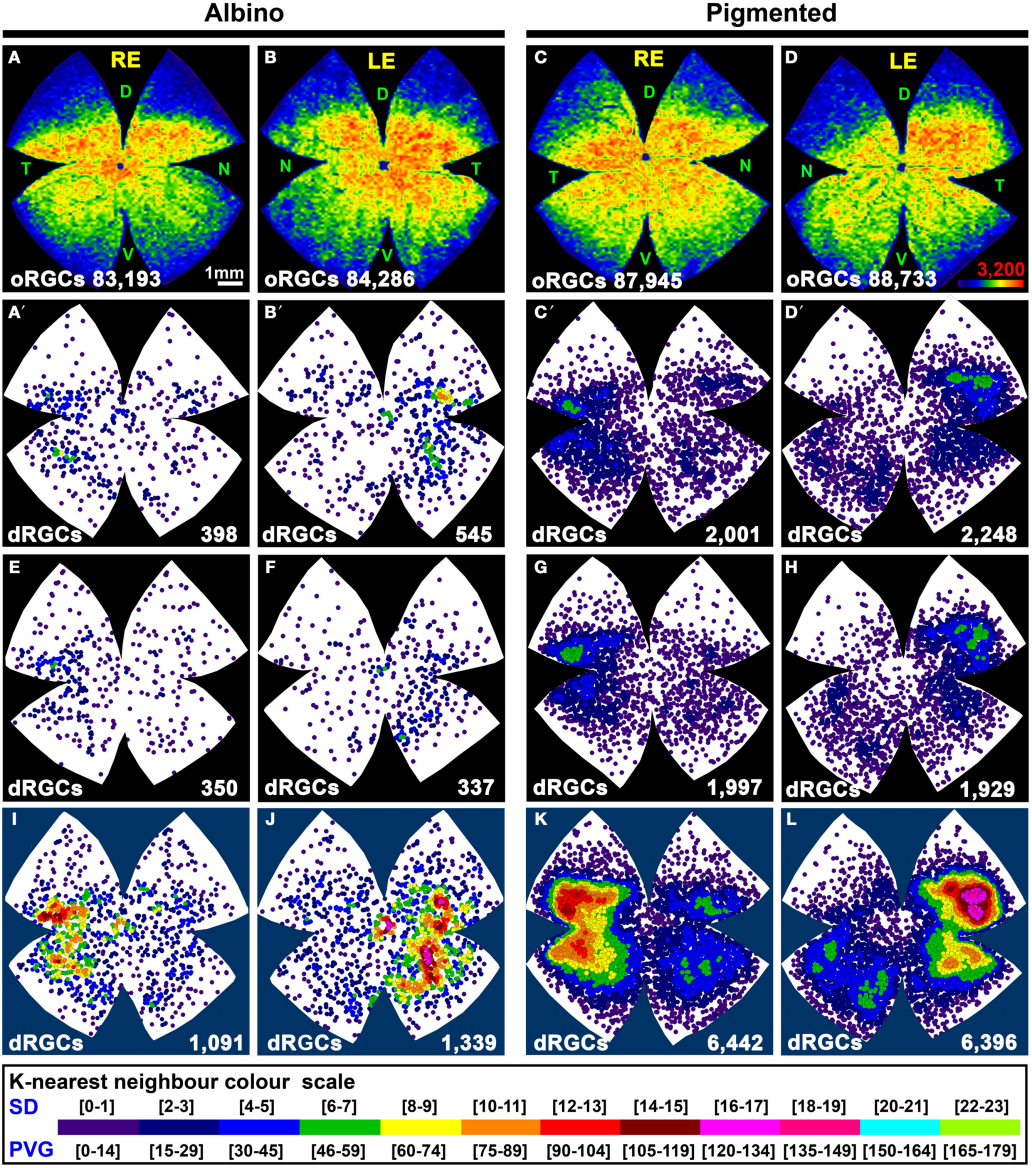
**Distribution of the total population of oRGCs and dRGCs in albino (SD) and pigmented (PVG) rats**. This study was done using retinas traced with FG from the optic nerve to identify the whole retinofugal projection. Two left columns: albino strain. Two right columns: pigmented strain. **(A–D)** Isodensity maps from a representative right **(A,C)** and left **(B,D)** retina showing the distribution of oRGCs. Color scale is shown in **(D)** bottom right, and ranges from 0 oRGCs/mm^2^ (purple) to 3200 or more oRGCs/mm^2^ (red). **(A′–D′)** Neighbor maps depicting the distribution of dRGCs in the same four representative retinas as **(A–D)**. **(E–H)** Neighbor maps depicting the distribution of dRGCs in other four retinas. At the bottom of each isodensity or neighbor map is shown the total number of oRGCs or dRGCs counted in the retina wherefrom the map has been generated. **(I–L)** dRGCs normalized neighbor maps; each one has been generated with data from 3 right **(I,K)** and 3 left **(J,L)** retinas. At the bottom of each normalized map is shown the total number of dRGCs represented. Color scale of neighbor maps is different for each strain because dRGCs are more abundant in the pigmented rat. RE, right eye; LE, left eye. Each pair of maps from a right and a left retina is from the same albino or pigmented animal. D, dorsal; T, temporal; N, nasal; V, ventral. Scale bar in **(A)**.

To identify the whole retinofugal projection, FG was applied around the optic nerve. As observed in Table 2 and Figure 4, there are significantly more dRGCs in the pigmented than in the albino rat (*T*-test *p* < 0.001). In fact, the pigmented strain has 5 times more dRGCs than the albino. In these same retinas, oRGCs were also quantified and the data show, in agreement with previous reports (Salinas-Navarro et al., 2009c; Nadal-Nicolás et al., 2012), that their number is also significantly higher in the pigmented than in the albino strain.

The sum of both populations (oRGCs + dRGCs) represents the total number of RGCs in both strains. Considering this number 100%, dRGCs amount for ~0.5 and 2.5% of the total RGC population, in the albino and pigmented strain, respectively (Table 2).

**Figure 5:**
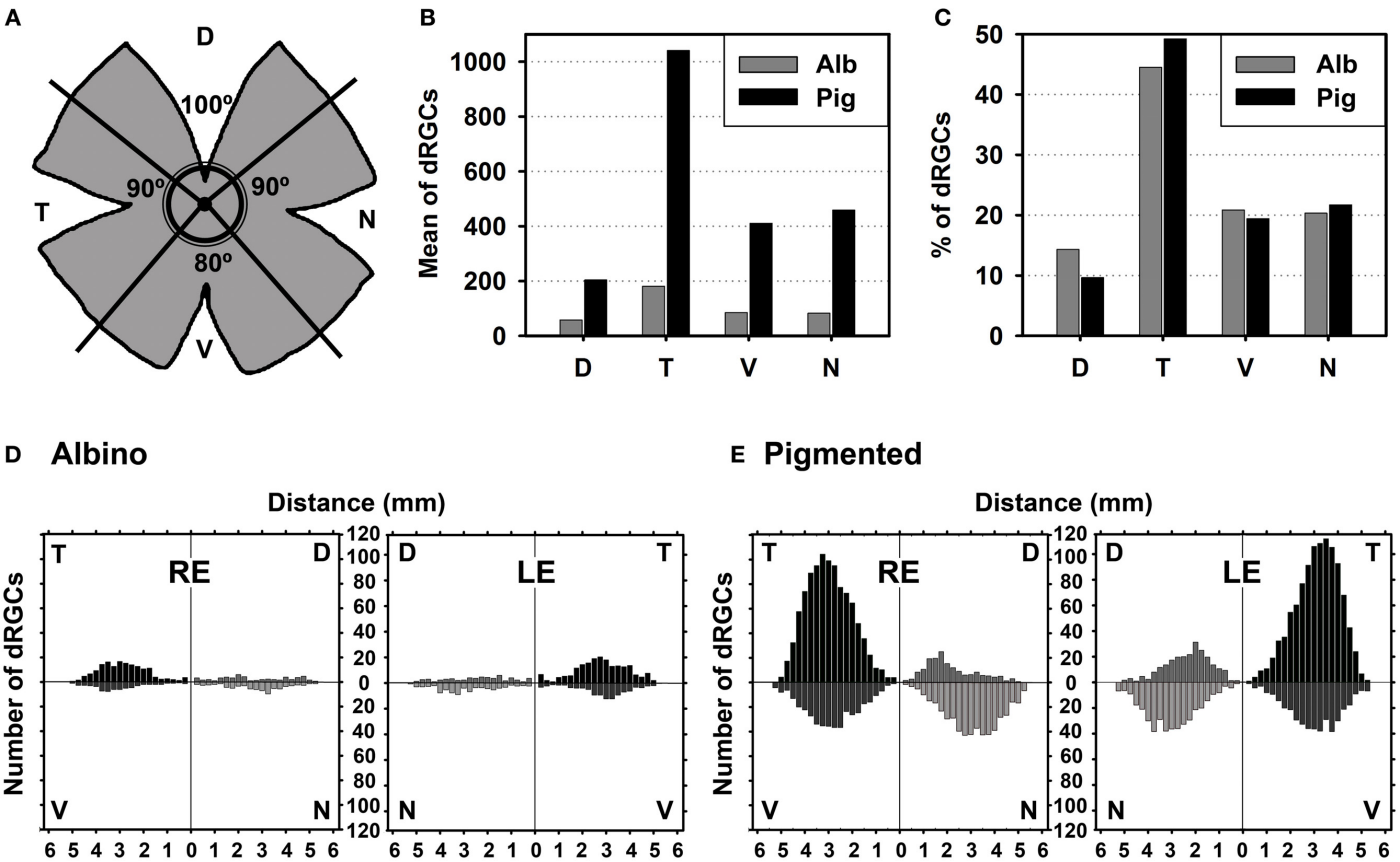
**dRGCs are more abundant in the temporal quadrant. (A)** Drawing showing the retinal division into its four retinal quadrants (D, dorsal; V, ventral; N, nasal; T, temporal). **(B)** Mean number of dRGCs per retinal quadrant in both rat strains. **(C)** Percentage of dRGCs per retinal quadrant in both rat strains. The total averaged number of dRGCs in each strain was considered 100%. **(D,E)** Graphs showing the mean number of dRGCs at a given distance from the optic nerve (ON) per retinal quadrant in the albino **(D)** and pigmented **(E)** rats. This analysis was done on the retinas normalized in Figures 4I–L (*n* = 6 retinas/strain, 3 left and 3 right). RE, right eye, LE, left eye.

In both rat strains, the topography of the dRGCs is similar (Figures 4, 5), and it is more easily seen in the normalized maps (Figures 4I–L, maps with blue background) and in the pigmented strain, because in this strain there are more dRGCs.

dRGCs are denser in a vertically oriented elliptical area situated in the temporal retina (Figures 4A′–H). The normalized dRGC maps show that their peak density may lay in the superior part of this ellipse, in the superotemporal quadrant of the retina, where the density of o-RGCs, is also highest (compare isodensity maps in Figures 4A–D with their respective neighbor maps in Figures 4A′–D′) as shown before in adult albino and pigmented rats (Nadal-Nicolás et al., 2009, 2012; Salinas-Navarro et al., 2009c).

In both strains dRGCs occupy, mainly, the central and equatorial retina along the naso-temporal axis leaving a void around the optic nerve. dRGCs are more abundant in the temporal than in the nasal retina (Figures 4A–L, 5). In fact, analyses of the number of dRGCs per quadrant (see Materials and Methods) show that the temporal retina bears almost 50% of the dRGCs while the dorsal retina holds ~10% of them, and the ventral and nasal quadrants equally share the remaining 40% (Figures 5A–C). These percentages are maintained in both strains in spite of the fact that albino rats have 5 times less dRGCs than pigmented rats.

dRGCs are placed between 0.5 and 5 mm from the optic nerve, and their maximum density is around 3 mm from the optic disk in both strains. (Figures 5D,E), except in the dorsal quadrant, where they peak at 2 mm.

### dRGCs projecting to both superior colliculi

Next, RGCs were quantified in retinas traced with FG from both SC which is the target region in the brain receiving a massive RGC input in rodents (Linden and Perry, 1983; Salinas-Navarro et al., 2009b,c). In agreement with these works, our data show that 88% of dRGCs and 97.5% of oRGCs in albino, and 100% of dRGCs and 97.6% of oRGCs in pigmented rats project to one or both SC (Table 2).

**Figure 6:**
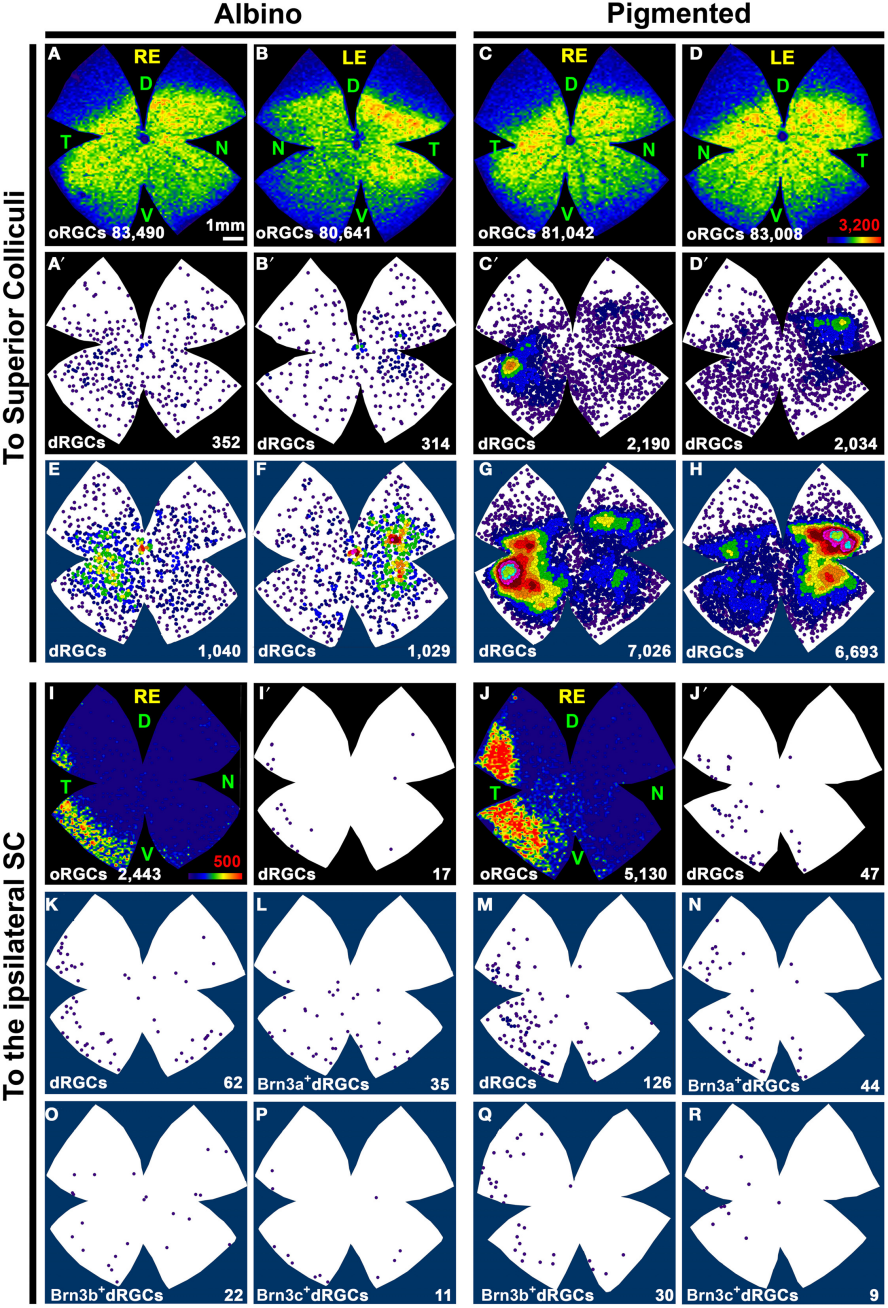
**Distribution of dRGCs and oRGCs projecting to both superior colliculi and to the ipsilateral colliculus. (A–D)** Isodensity maps showing the distribution of oRGCs traced from both SC. **(A′–D′)** Neighbor maps showing the distribution of dRGCs traced from both SC in the same retinas as in **(A–D)**. **(E–H)** Neighbor maps generated with data from 3 retinas each, showing the normalized distribution of dRGCs traced from both SC. **(I,J)** Isodensity maps showing the distribution of ipsilateral oRGCs. **(I′,J′)** Neighbor maps showing the distribution of ipsilateral dRGCs in the same retinas as **(I,J)**. **(K,M)** Neighbor maps generated with data from 3 retinas each, showing the normalized distribution of ipsilateral dRGCs. **(L,N–R)** Neighbor maps generated with data from 3 retinas each, showing the normalized distribution of ipsilateral dRGCs that express Brn3a **(L,N)**, Brn3b **(O,Q)** or Brn3c **(P,R)**. Color scale for the isodensity maps is shown in **(D,I)** bottom right and is adjusted to each RGC population, it goes from 0 RGCs/mm^2^ (purple) to 3200 **(A–D)** or 500 **(I,J)** or more RGCs/mm^2^ (red). Neighbor maps color scale is the same as in Figure 4. At the bottom of each map is shown the total number of oRGCs/dRGCs represented. RE, right eye; LE, left eye. Each pair of maps from a right and a left retina is from the same albino or pigmented animal. Maps representing ipsilateral RGCs are from right retinas D, dorsal; V, ventral; N, nasal; T, temporal. Bar scale in **(A)**.

As expected from the quantitative data, the distribution of dRGCs/oRGCs projecting to the SC (Figures 6A–H) is the same as that observed when they are traced from the optic nerve.

### Ipsilateral dRGCs

To identify the ipsilateral projection, the tracer was applied only onto the right SC and the dRGCs/oRGCs were counted in the right retinas (Table 2). In accordance with previous works (Lund, 1965; Nadal-Nicolás et al., 2012), ipsilateral oRGCs are more abundant in the pigmented than in the albino rat and their percentage amounts for 4.3 and 2.4% of the SC projection, respectively. Thus, the majority of the oRGCs send their projections to the contralateral superior colliculus. This is also the case for the dRGCs. However, and in contrast with mice (Dräger and Olsen, 1980) the percentage of dRGC ipsilaterality is significantly higher (Mann Whitney test *p* < 0.05) in the albino (4.2%) than in the pigmented strain (2.2%).

The distribution of ipsilateral oRGCs is shown in Figures 6I,J and concords with previous reports (Lund, 1965; Dräger and Olsen, 1980; Nadal-Nicolás et al., 2012). They are mostly located in the peripheral infero-temporal retina in the shape of a crescent moon. Ipsilateral dRGCs are also located in the infero-temporal peripheral retina (Figures 6I′,J′,K,M).

### dRGCs and expression of Brn3 transcription factors

Recently, we have published that 96% of the adult rat oRGCs express Brn3a, 65.5% Brn3b and 51% Brn3c (Nadal-Nicolás et al., 2012), in agreement with data shown here in Table 3.

**Table 3:** **Number of oRGCs and dRGCs that express Brn3 factors**.

		**Albino**		**Pigmented**	
		**dRGCs**	**oRGCs**	**dRGCs**	**oRGCs**
Total	Brn3a^†^ (*n* = 18–19/strain)	362 ± 30	80,974 ± 2025	2030 ± 282	83,903 ± 1937
	Total (displaced + orthotopic)	81,251 ± 1910		85,903 ± 2065	
	Brn3b (*n* = 6–8/strain)	201 ± 45	53,861 ± 1680	925 ± 130	55,513 ± 3803
	Total Brn3b (displaced + orthotopic)	54,062 ± 1687		56,434 ± 3930	
	Brn3c (*n* = 6–8/strain)	186 ± 49	42,030 ± 2588	885 ± 128	41,838 ± 2509
	Total (displaced + orthotopic)	42,216 ± 2599		42,723 ± 2551	
	Brn3 (*n* = 6–8/strain)	357 ± 25	84,632 ± 2208	2143 ± 300	83,646 ± 2789
	Total (displaced + orthotopic)	85,057 ± 2152		85,789 ± 2746	
Ipsilateral	FG and Brn3a^+^ (*n* = 3–5/strain)	10 ± 4	864 ± 27	15 ± 8	1354 ± 60
	FG and Brn3b^+^ (*n* = 3–4/strain)	7 ± 1	498 ± 41	10 ± 4	782 ± 29
	FG and Brn3c^+^ (*n* = 3–4/strain)	4 ± 2	213 ± 25	3 ± 2	251 ± 16

*Mean ± standard deviation of oRGCs and dRGCs that express Brn3a, Brn3b, Brn3c, or Brn3 (triple immunodetection with the same fluorophore). To quantify the number of ipsilateral oRGCs/dRGCs that express each of these transcription factors, only those FG^+^ neurons traced from the ipsilateral colliculus were taken into account*.

† *The number of Brn3a^+^RGCs is the average number counted in the groups traced from the ON, both SC, and double immunodetected with melanopsin (see Table 1 for experimental design)*.

Next, we next sought to determine the number and location of dRGCs that express each Brn3 member and to assess whether this expression was similar to that found in oRGCs.

**Figure 7:**
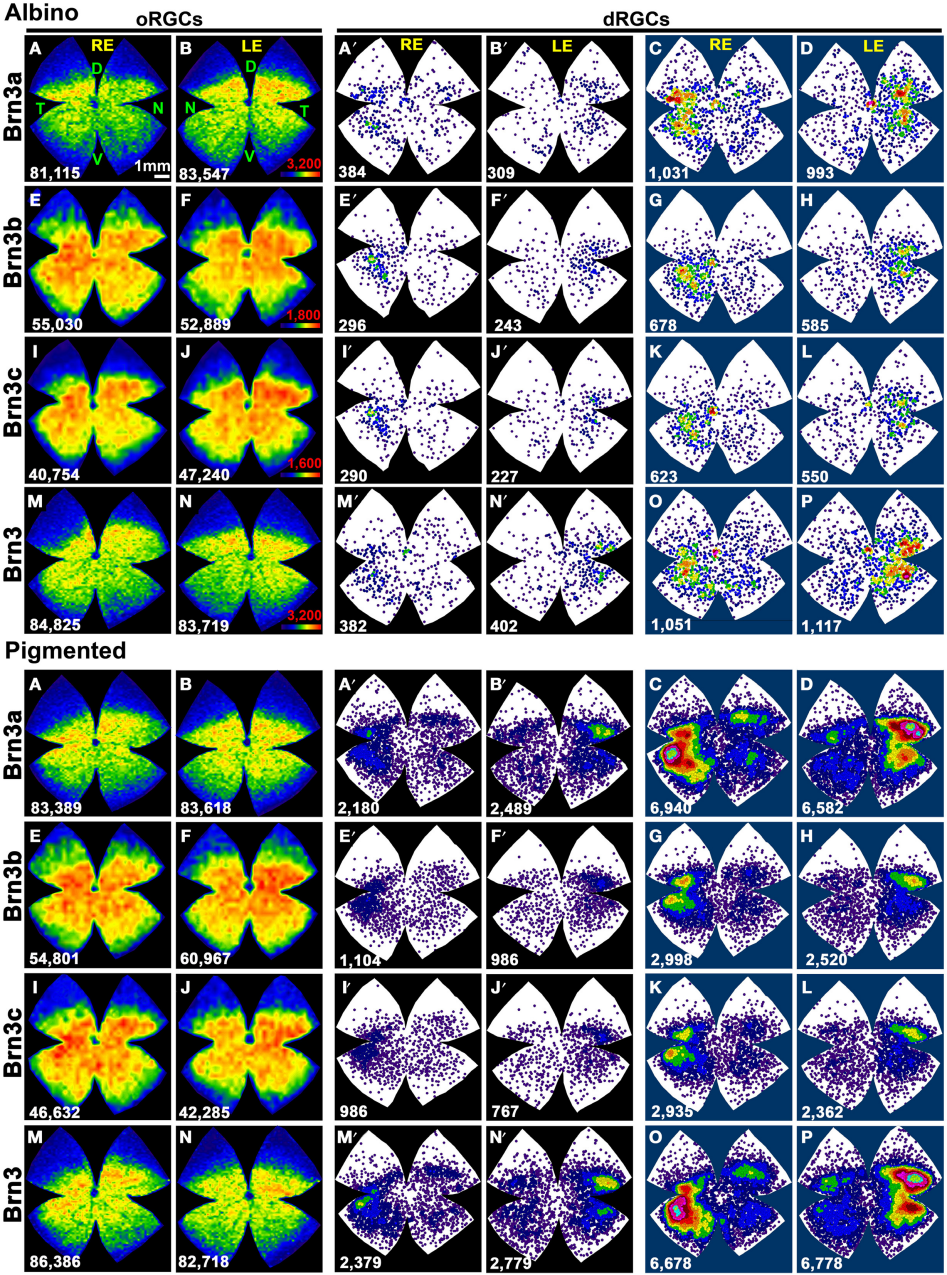
**Distribution of Brn3 positive oRGCs and dRGCs in albino and pigmented rats**. *Two leftmost columns*: isodensity maps showing the distribution of Brn3a **(A,B)**, Brn3b **(E,F)**, Brn3c **(I,J)**, and Brn3 **(M,N)** positive oRGCs. The number of RGCs counted for each map is shown at its bottom. Color scale is adjusted to each RGC population (in **B,F,J,N** bottom right), and ranges from 0 (purple) RGCs/mm^2^ to 3200 (Brn3 or Brn3a), 1800 (Brn3b), 1600 (Brn3c) or more RGCs/mm^2^, respectively. *Two middle columns*: neighbor maps depicting the distribution of Brn3a **(A′,B′)**, Brn3b **(E′,F′)**, Brn3c **(I′,J′)** and Brn3 **(M′,N′)** positive dRGCs Notice that isodensity and neighbor maps labeled with the same letter are from the same retina (**A–A′**, **B–B′** etc.). *Two rightmost columns*: Normalized neighbor maps where dRGCs from. three retinas are represented. **(C,D)** Brn3a^+^; **(G,H)** Brn3b^+^; **(K,L)** Brn3c^+^; **(O,P)** Brn3^+^. The total number of dRGCs represented in each neighbor map is shown at its bottom. Color scale for neighbor maps is the same shown in Figure 4. RE, right eye; LE, left eye. Each pair of maps from a right and a left retina is from the same albino or pigmented animal. D, dorsal; V, ventral; N, nasal; T, temporal. Bar scale: 1 mm.

Table 3 shows the total number of dRGCs and oRGCs that express each of the three Brn3 members or that express any Brn3 isoforms (triple immunodetection using the same fluorophore). In spite of the fact that Brn3b and Brn3c are expressed by less than 50% of the dRGCs, their distribution (Figures 7A–D: Brn3a, Figures 7E–H: Brn3b, Figures 7I–L: Brn3c, and Figures 7M–P: Brn3) resembles the distribution of the whole dRGC population (Figures 4, 6A–H).

Brn3a immunodetection labels 88 and 91% of the FG^+^dRGCs in the albino and pigmented strain, respectively (Table 3). Because of this fact and because the distribution of Brn3a^+^dRGCs is comparable to the distribution of dRGCs identified by tracing from the optic nerve (compare maps if Figures 7A′,B′,C,D with maps in Figures 4A′–H), we conclude that Brn3a is a good tool to identify dRGCs.

The mean number of Brn3a^+^dRGCs does not differ from the total number of Brn3^+^dRGCs and this means that all the dRGCs that express a Brn3 member, express Brn3a. Brn3b is expressed by 47 or 40% of dRGCs in the albino and pigmented strain respectively, while Brn3c is expressed by 44% (SD) and 38% (PVG) of them. These percentages are slightly lower than in the oRGC population (Nadal-Nicolás et al., 2012).

Because in all traced retinas Brn3a was immunodetected, it was possible to assess the correspondence between FG-tracing and Brn3a expression in the dRGC population (Table 4). When traced with FG from the optic nerve, all Brn3a^+^dRGCs were also FG^+^. However, there was an 11% (albino) and a 9% (pigmented) of the dRGCs that were FG^+^ but Brn3a^−^. All these cells corresponded to melanopsin^+^ dRGCs (see below).

**Table 4:** **Correspondence between fluorogold tracing and Brn3a immunodetection in the dRGC population**.

**Labeling**	**dRGCs**	
	**Albino (*n* = 6)**	**Pigmented (*n* = 7)**
FG^+^Brn3a^+^	373 ± 76	2084 ± 200
FG^+^ Brn3a^−^	49 ± 6	209 ± 9
FG^−^Brn3a^+^	None	None
Total FG	422 ± 80	2293 ± 197
Total Brn3a	373 ± 76	2084 ± 200

Data on this table are from the group of animals traced from the optic nerve. All Brn3a^+^ dRGCs were FG^+^, but some FG^+^dRGCs were Brn3a^−^. These are the intrinsically photosensitive dRGCs, m-dRGCs (see Table 5 and results).

With respect to the ipsilateral dRGCs, (Table 3), Brn3a is expressed by 55 and 30% of the albino and pigmented dRGCs, respectively. Brn3b and Brn3c are expressed by 38 and 22% (albino) and 20 and 6% (pigmented) of them, respectively. The normalized distribution of ipsilateral dRGCs that express Brn3a, Brn3b, or Brn3c is shown in Figures 6L,N (Brn3a), Figures 6O,Q (Brn3b), and Figures 6P,R (Brn3c).

### dRGCs that express melanopsin (m-dRGCs)

Within the melanopsin population, 2.3% in albino and 8.7% in pigmented rats were displaced (Table 5). These percentages are significantly higher (Mann Whitney test *p* = 0.002) than the proportion of non-melanopsin RGCs (Brn3a^+^) that are displaced (0.43% albino and 2.24 pigmented). As occurs with non-melanopsin dRGCs, there are significantly more m-dRGCs in the pigmented than in the albino strain (Mann-Whitney test *p* < 0.001).

**Table 5:** **Number of intrinsically photosensitive displaced retinal ganglion cells (m-dRGCs) and intrinsically photosensitive orthotopic retinal ganglion cells (m-oRGCs) in albino and pigmented rats**.

		**Albino**		**Pigmented**	
		**m-dRGCs**	**m-oRGCs**	**m-dRGCs**	**m-oRGCs**
A: Total	Melanopsin (*n* = 6/strain)	54 ± 12	2329 ± 198	212 ± 21	2216 ± 154
	mRGC population (displaced + orthotopic)	2383 ± 178		2428 ± 172	
B: Ipsilateral	FG^+^ and melanopsin^+^ (*n* = 3–5/strain)	0.2 ± 0.4	64 ± 14	12 ± 4	89 ± 8
	Total ipsilateral m-RGCs (displaced + orthotopic)	65 ± 14		101 ± 8	

***A**: Mean number ± standard deviation of displaced and orthotopic m-RGCs*.

***B**: Mean number ± standard deviation of displaced and orthotopic m-RGCs that project to the ipsilateral colliculus*.

The proportion of dRGCs that express melanopsin is 13% (albino) and 9.3% (pigmented), a much higher percentage than in the orthotopic population (~2.6% in both strains) (Tables 2, 5).

The soma diameter of m-oRGCs in both strains ranges from 10 to ~21 (Table 6). Their mean soma diameter is 12.3 ± 1.8 μm in albino and 15.0 ± 1.8 μm in pigmented, which may reflect a higher proportion of large m-oRGCs in the pigmented rat.

**Table 6:** **Soma diameter of m-dRGCs and m-oRGCs in albino and pigmented rats**.

		**Albino**		**Pigmented**	
		**m-dRGCs *n* = **73****	**m-oRGCs *n* = **381****	**m-dRGCs *n* = **117****	**m-oRGCs *n* = **333****
Soma diameter (μm)	Mean ± SD	12.5 ± 1.5	12.3 ± 1.8	12.0 ± 1.4	15.0 ± 1.8
	Max	15.4	21.6	15.8	20.5
	Min	9.5	10.5	8.0	10.5

*Mean ± standard deviation (SD), maximum (Max) and minimum (Min) soma diameter (μm) of m-RGCs (average of 12 samples/retina, n = 4 retinas per strain), n: total number of mRGCs analyzed*.

With respect to m-dRGCs, their average size does not differ much from that of m-oRGCs in albino rats, while in pigmented animals it is slightly smaller. However, in both strains some m-dRGCs are littler than the smaller m-oRGC since some m-dRGCs have a minimum soma diameter 1 (albino) or 2 (pigmented) μm shorter than the minimum m-oRGC diameter. In addition, the maximum size of m-dRGCs is 15 μm, this may mean that all types of m-oRGCs are represented in the displaced population only that they are smaller and/or that the biggest m-RGCs (M4, Estevez et al., 2012) are not represented in the displaced population.

**Figure 8:**
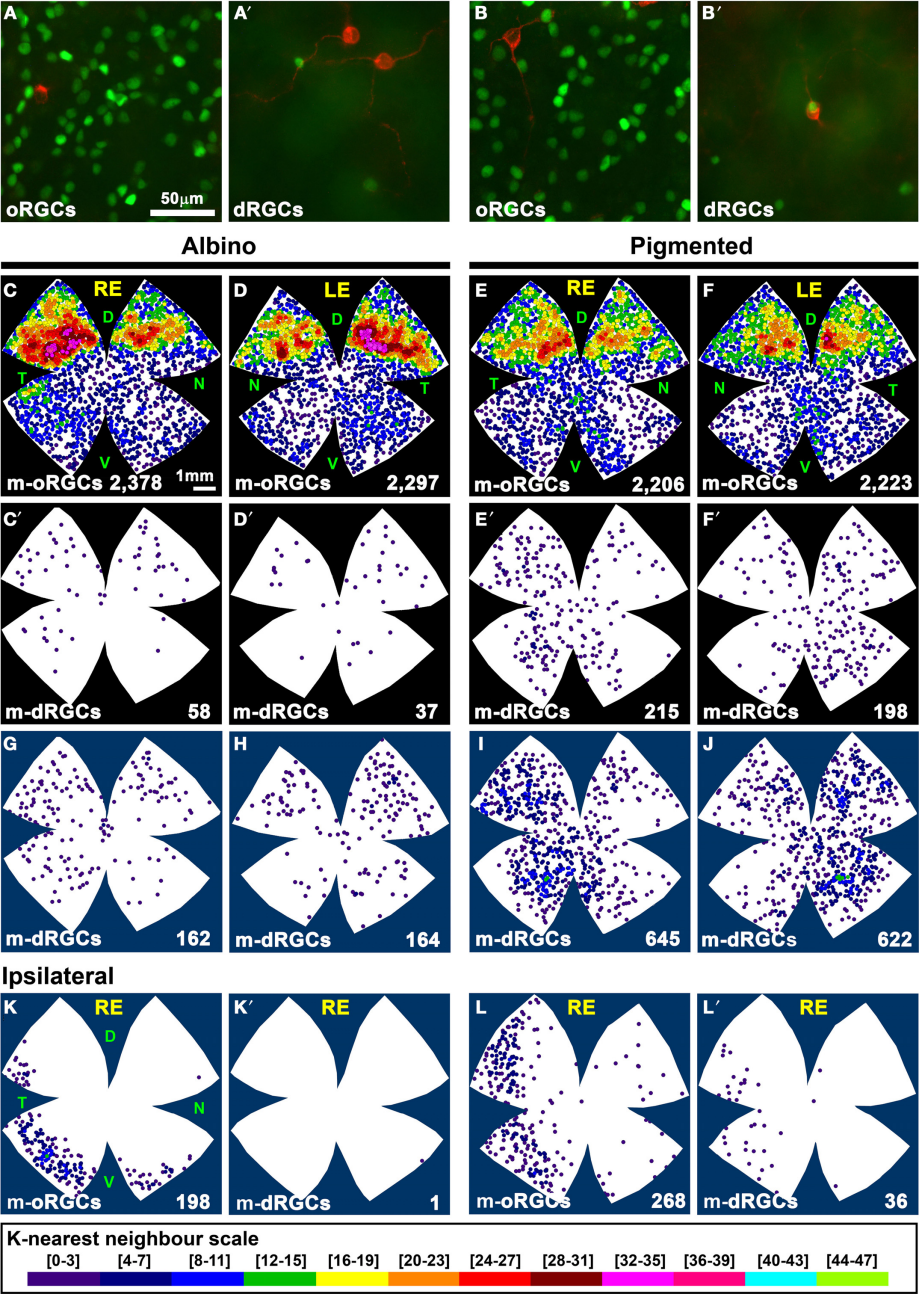
**Distribution of the total and ipsilateral population of m-dRGCs and m-oRGCs in albino and pigmented rats. (A–B′)** Magnifications from two areas **(A,B)** of a pigmented flat mounted retina double immunodetected for Brn3a (green) and melanopsin (red). **(A,B)** oRGCs. **(A′,B′)** dRGCs. In **(B′)** is shown one of the few m-dRGC that were also Brn3a^+^. **(C–F)** Neighbor maps showing the distribution of m-oRGCs in albino **(C,D)** and pigmented **(E,F)** rat retinas. **(C′–F′)** neighbor maps showing the distribution of m-dRGCs in the same retinas as **(C–F)**. **(G–J)** Normalized neighbor maps where m-dRGCs from three retinas are represented. **(K,L)** Normalized neighbor maps where ipsilateral m-oRGCs from three retinas are represented. **(K′,L′)** Normalized neighbor maps where ipsilateral m-dRGCs from the same three retinas as **(K,L)** are represented. The total number of m-RGCs represented in each neighbor map is shown at its bottom. Color scale for neighbor maps is shown at the bottom of the figure, and it is the same for both strains. RE, right eye; LE, left eye. Each pair of maps from a right and a left retina is from the same albino or pigmented animal. Maps representing ipsilateral mRGCs are from right retinas. D, dorsal; V, ventral; N, nasal; T, temporal. Bar scales in **(A,E)**.

We have recently documented that only a 0.23% of the population of m-oRGCs in rats express Brn3a (Nadal-Nicolás et al., 2012; Galindo-Romero et al., 2013a) (Figures 8A–B′). Out of the total number of m-dRGCs counted, only 5 and 11 were Brn3a^+^ in albino and pigmented rats, respectively. Thus, the population of m-dRGCs are the dRGCs identified by tracing from the optic nerve that were Brn3a^−^ (49 and 209 cells, Tables 3, 4).

The distribution of m-oRGCs and m-dRGCs is shown in Figure 8. In the albino strain m-dRGCs are found predominantly in the dorsal-retina, in the region where m-oRGCs are more abundant in both strains (Figures 8C,D,C′,D′,I,J). In the pigmented rat, m-dRGCs are mainly located in the hemitemporal retina (Figures 8E,F,E′,F′,K,L).

There are significantly more ipsilateral m-oRGCs in the pigmented than in the albino strain (Table 5, Figures 8K,L) and they are found in the temporal retinal crescent (see Figures 6I,J). Only 1 ipsilateral m-dRGC was found in all the albino retinas (Figure 8K′), and a mean of 12 per retina in the pigmented strain (Figure 8L′). Thus, in contrast with ipsilateral non-melanopsin dRGCs, the percentage of ipsilateral m-dRGCs is significantly higher (Mann Whitney test *p* < 0.05) in the pigmented than in the albino strain.

### Response to injury: optic nerve transection and ocular hypertension

Are dRGCs more resilient to injury than oRGCs? To answer this question, both populations were quantified 7 days after ONT or 14 days after OHT. Data in Table 7 show that the proportion of dRGCs and oRGCs that survive is similar within each insult, indicating that the susceptibility of dRGCs is similar to that of oRGCs.

**Table 7:** **Survival of oRGCs and dRGCs after unilateral left optic nerve transection or ocular hypertension in albino rats**.

			**Albino**			
			**Left retinas**		**Naïve retinas**	**Right retinas**
			**dRGCs**	**oRGCs**	**dRGCs**	**oRGCs**
ONT 7dpl *n* = 8	Brn3a	Mean ± SD	184 ± 45	38,238 ± 7152	362 ± 30	79,140 ± 1920
		% of control	51%	48%	–	–
OHT 14dpl *n* = 20	Traced	Mean ± SD	69 ± 98	14,177 ± 18,726	375 ± 37	80,912 ± 3343
		% of control	18%	17%	–	–
	Brn3a	Mean ± SD	172 ± 73	38,111 ± 14,616	362 ± 30	82,731 ± 3587
		% of control	47%	46%	–	–

Mean number ± standard deviation and percentage of surviving oRGCs and dRGCs 7 days after ONT or 14 days after OHT. After both lesions, survival was assessed by Brn3a immunodetection In the OHT-group RGCs were also traced with FG form the superior colliculus to show the extension of the lesion. As control for oRGCs, traced and/or Brn3a^+^ oRGCs were counted in the right contralateral eyes, while the control population of dRGCs was obtained from naive retinas (Table 2). dpl: days post-lesion.

**Figure 9:**
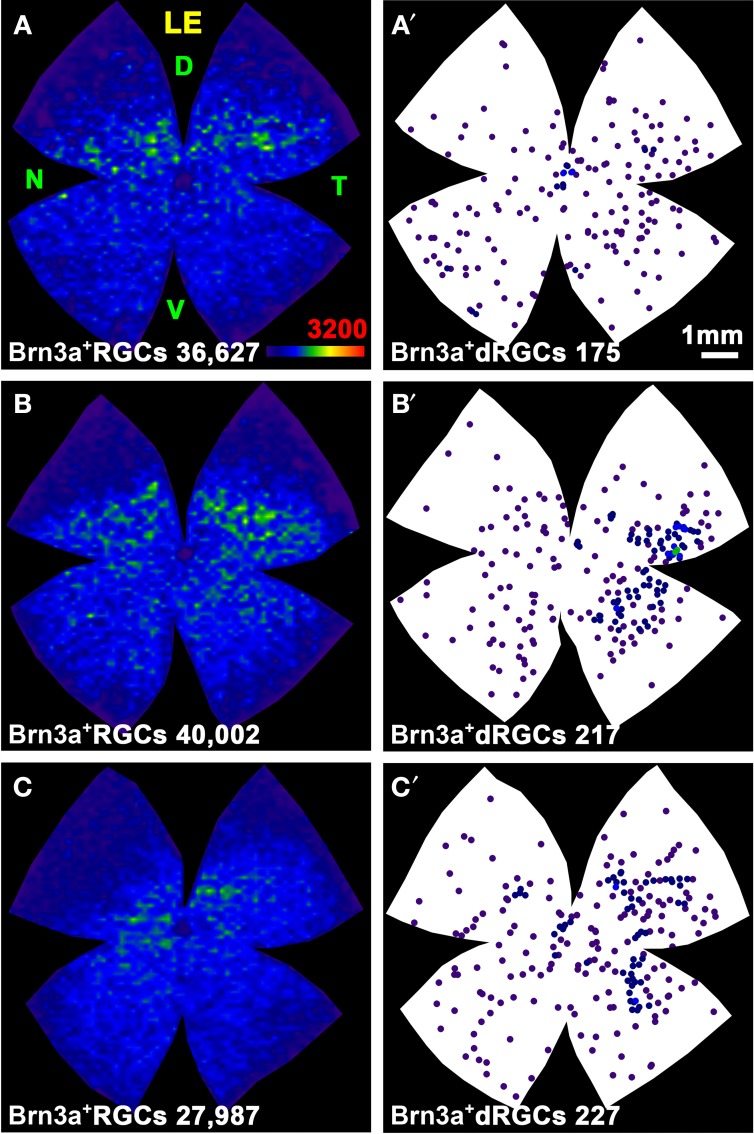
**Distribution of surviving oRGCs and dRGCs after ONT. (A–C)** Three representative isodensity maps showing the distribution of surviving Brn3a^+^oRGCs 7 days after intraorbital nerve transection (ONT). **(A′–C′)** Neighbor maps from the same retinas showing the distribution of surviving Brn3a^+^dRGCs. At the bottom of each representation is shown its number of oRGCs or dRGCs represented. Color scale for isodensity maps in A bottom right, for neighbor maps see Figure 4. LE, left eye; D, dorsal; V, ventral; N, nasal; T, temporal. Bar in **(A′)**.

**Figure 10:**
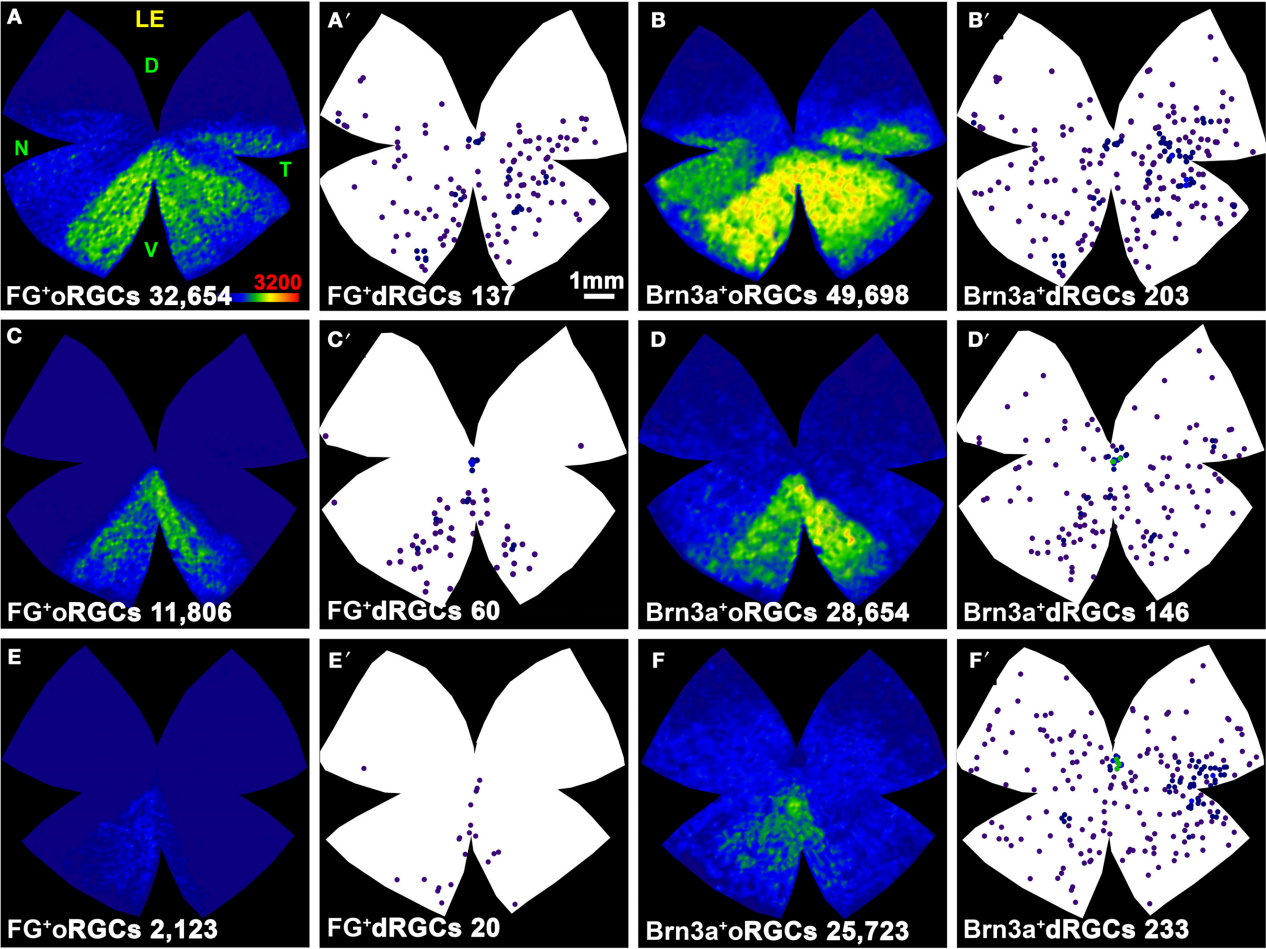
**Distribution of oRGCs and dRGCs after OHT**. Maps of three representative retinas (one per row) showing the distribution of traced oRGCs **(A,C,E)** and dRGCs **(A′,C′,E′)**, and of Brn3a^+^ oRGCs **(B,D,F)** or dRGCs **(B′,D′,F′)** 14 days after ocular hypertension. At the bottom of each map is shown its number of RGCs or dRGCs represented. Color scale for isodensity maps in A bottom right, for neighbor maps see Figure 4. LE, left eye; D, dorsal; V, ventral; N, nasal; T, temporal. Bar in **(A′)**.

ONT causes a diffuse and homogeneous loss of dRGCs and oRGCs (Figure 9) and OHT a sectorial loss (Figure 10) (Salinas-Navarro et al., 2010; Vidal-Sanz et al., 2012). To assess RGC survival, Brn3a was immunodetected (Figures 10B,B′,D,D′,F,F′). These maps show that Brn3a^+^ dRGCs/oRGCs are densest in the areas of tracing, paralleling the densities of the FG-traced ones. In addition, at this time post-OHT Brn3a^+^ dRGCs/oRGCs are still observed in the retinal areas devoid of FG-traced neurons, i.e., at 14 days post-induction of the OHT, in the areas where axonal transport is impaired there still are surviving dRGCs/oRGCs, in agreement with previous reports (Salinas-Navarro et al., 2009a, 2010; Cuenca et al., 2010; Vidal-Sanz et al., 2012).

## Discussion

In this work we provide new data regarding the number, distribution, Brn3 and melanopsin expression, and projection to the contralateral or ipsilateral superior colliculus of orthotopic and displaced RGCs.

We have studied these cells in albino and pigmented rats because albinism causes a long list of abnormalities in the visual system such as an impaired visual acuity and optokinetic nystagmus, defects in the crossing of the retinofugal projections (Lund, 1965; Dräger and Olsen, 1980; Balkema et al., 1981; Dreher et al., 1985; Mangini et al., 1985; Dräger and Balkema, 1987; Pak et al., 1987; Balkema and Dräger, 1990, 1991; Prusky et al., 2002; Nadal-Nicolás et al., 2012), lower number of oRGCs (Salinas-Navarro et al., 2009c; Nadal-Nicolás et al., 2012) and of rods and rhodopsin levels, and, in mice, changes in the topographic expression of the S-opsin (Applebury et al., 2000; Jelcick et al., 2011; Ortín-Martínez et al., 2014) and a decrease in the number of dRGCs (Dräger and Olsen, 1980; Balkema and Dräger, 1990).

Because oRGCs and dRGCs were studied in the same retinas, here we show for each of the analysis performed a complete topographic and quantitative study of the RGC population (oRGCs + dRGCs) in both rat strains. To avoid repetition, we will discuss the whole retinofugal projection (all RGCs and the melanopsin^+^ subtype) and then the discussion will be focused on the dRGCs. However, it is important to highlight that we report as well the total number and distribution of: i/ ipsilateral RGCs *per se* or expressing each Brn3 member or melanopsin, ii/ RGCs projecting to both superior colliculi and, iii/ RGCs expressing each of the Brn3 transcription factors or expressing any of them (Brn3^+^).

### Total number of RGCs

The population of RGCs in rats and mice has been thoroughly studied by our group in control and injured retinas (Nadal-Nicolás et al., 2009, 2012; Parrilla-Reverter et al., 2009a,b; Salinas-Navarro et al., 2009a,b,c, 2010; García-Ayuso et al., 2010; Ortín-Martínez et al., 2010; Galindo-Romero et al., 2011, 2013a,b; Sánchez-Migallón et al., 2011). In those previous studies only orthotopic RGCs were considered. Using retinas traced from the optic nerve, thus labeling the whole RGC population, we counted oRGCs and dRGCs thus reporting for the first time their total number in rats (see Tables 2, 5). In agreement with our previous reports (Salinas-Navarro et al., 2009c; Nadal-Nicolás et al., 2012), the pigmented rat has a significantly higher number of RGCs.

Although the total RGC population (oRGCs + dRGCs) in albino rats is only 7%, smaller, this difference is mostly caused by a five-fold reduction of dRGCs. The effect of albinism on the number of dRGCs has been also observed in mice, but the reduction in this species is smaller (2.4-fold) (Dräger and Olsen, 1980; Balkema and Dräger, 1990).

### Melanopsin expression: intrinsically photosensitive RGCs

Melanopsin^+^RGCs are RGCs whose function is mostly, but not only, ascribed to non-forming image vision (Brown et al., 2012, for review see: Schmidt et al., 2011; Pickard and Sollars, 2012).

In mice, most of the displaced melanopsin^+^RGCs resemble the M1 subtype (Pires et al., 2009; Berson et al., 2010), and few are M2-like. Here we have not attempted to discriminate both subtypes, but rather to describe the number and topography of all melanopsin^+^RGCs, orthotopic and displaced. The soma diameter of m-RGCs is an indicator of their subtype (M1 < M < M3 < M4; Schmidt et al., 2011; Estevez et al., 2012; Sand et al., 2012). The size of the different mRGC subtypes in mice varies among reports (reviewed in Sand et al., 2012), and so M1 mRGCs range between 13.9 and 17 μm, M2 between 15 and 22 μm, M3 are around 17 μm and M4 between 17 and 22 μm.

In this work we show that, in rat, melanopsin^+^oRGCs have a soma size from 10 μm to 21 (albino) or 20 (pigmented) μm. In the pigmented strain, however, the mean m-oRGC soma diameter is larger than in albinos (15 vs. 12.3 μm), which suggests a higher proportion of large m-oRGCs in the pigmented rat.

With respect to m-dRGC, they are smaller than their orthothopic counterpart, since their minimum diameter is shorter (9.5 μm albino and 8 μm in pigmented). In addition, their maximum diameter is 15 μm, which indicates that the biggest subtype(s) of melanopsin^+^RGCs (M4 and maybe M3) may not be represented in the displaced population.

The number of m-oRGCs does not differ between albino and pigmented animals. Our data from albino rats agrees with a previous report from our laboratory (Galindo-Romero et al., 2013a) and others (Hattar et al., 2002). In pigmented rats, Vugler et al. (2008) studied the population of m-oRGCs and observed, in agreement with this work, a higher number of them in the dorsal retina. Here, we add to these data their total population and detailed topography.

We describe in this article, as documented before (Nadal-Nicolás et al., 2012; Galindo-Romero et al., 2013a), that rat mRGCs do not express Brn3a. In both rat strains, m-oRGCS and Brn3a^+^oRGCs are distributed in a complementary fashion. However, there are subtle differences among them: in the albino rat their higher densities are found in the dorsal retina (Hannibal et al., 2002; Hattar et al., 2002) peaking in the temporal quadrant, above the area of highest RGC densities (Galindo-Romero et al., 2013a, and Figures 8C,D). In the pigmented strain there are also more m-oRGCs in the dorsal retina, as reported (Vugler et al., 2008), except that their peak is not temporal but purely dorsal. Furthermore, in the ventral retina occurs the same, in albinos there are more in the temporal quadrant, while in the pigmented rat they are located in a vertical band.

The proportion of m-oRGCs that project to the ipsilateral SC is similar to non-melanopsin oRGCs (2.7% albino, 4% pigmented), and they are mostly observed in the retinal temporal crescent (see below).

In the albino rat retina the number of m-dRGCs is significantly lower (four-fold) than in the pigmented rat. Their topography does not follow the general dRGC distribution (see below), they are predominantly found in the dorsal retina of albino rats, and in the hemitemporal retina of pigmented ones.

To date is not known if m-dRGCs play a specific role among the whole battery of functions that m-RGCs display (Schmidt et al., 2011), but our anatomical data open the question as to whether albino animals show different melanopsin-driven responses than pigmented ones. Whether m-dRGCs are as well affected in albino mice has yet to be determined.

Ipsilateral m-dRGCs are extremely rare in the albino retina (we have found only 1 cell in 5 retinas). In pigmented rats ipsilateral m-dRGCs are scarce and amount approximately for 5.6% of the m-dRGCs, and for 12% the ipsilateral m-RGCs. They are found as well in the ipsilateral retinal crescent.

In conclusion, albinism in rat produces in the melanopsin population the same defects than in the rest of RGCs: decreased number of displaced m-RGCs and a lower ratio of ipsilaterality.

### Expression of Brn3 factors

Brn3 factors are essential for the development, differentiation, morphology and function of RGCs (Isenmann et al., 2003; Badea et al., 2009; Badea and Nathans, 2011). In both rat strains, all of the oRGCs except half of the ipsilateral projection and those that are melanopsin^+^, express Brn3a (96%), while Brn3b and Brn3c are expressed by 65.5 and 51.5% of them, respectively (Nadal-Nicolás et al., 2012). Thus, Brn3a is an excellent marker to study in parallel, but separately, the general population of RGCs vs. melanopsin^+^RGCs (Galindo-Romero et al., 2013a).

In dRGCs the percents of Brn3 expression are maintained, although with respect to Brn3b and Brn3c are slightly lower than in the oRGC population. Because there are not significant differences between both strains, it seems that albinism does not affect the expression of Brn3 proteins.

The topography of Brn3a-, b- or c- positive dRGCs is roughly the same as of the total dRGC population, in spite of the fact that half of the dRGCs do no express Brn3b and Brn3c. The same is observed for oRGCs (here and Nadal-Nicolás et al., 2012). This indicates that, at least topographically, neither oRGCs nor dRGCs are segregated by the expression of a given Brn3 factor.

### dRGC projections

oRGCs in rats and mice project massively to the contralateral SC and the ipsilateral projection is greatly diminished in albino animals (Lund, 1965; Dräger and Olsen, 1980; Nadal-Nicolás et al., 2012). In agreement with those reports, we observed that 4 and 2.4% of the pigmented and albino oRGCs, respectively, project ipsilaterally.

In mice and rabbits dRGCs project to the SC (Dräger and Olsen, 1980, 1981; Vaney et al., 1981; Balkema and Dräger, 1990). Here we show that this is also the case in rats. Like oRGCs, most of dRGCs (~96–98%) project contralaterally. However, contrary to oRGCs, the percentage of ipsilateral dRGCs is higher in albinos (4.3%) than in pigmented (2.2%) rats. Furthermore, while in albinos ipsilateral dRGCs are proportionally more abundant than oRGCs, in pigmented the proportion of ipsilaterality in dRGCs is halved.

This situation differs from mice in two points: in mice the percentage of ipsilateral dRGCs is higher (13–21%) than of ipsilateral oRGCs (1%) (Dräger and Olsen, 1980) and pigmented animals have a higher proportion of ipsilateral dRGCs than albinos. Yet, considering absolute numbers pigmented animals have 2.3 times more ipsilateral dRGCs than albinos in both species (Table 2 here and Table 2 in Dräger and Olsen, 1980).

Finally, the population of dRGCs in pigmented rats is 5 times bigger than albinos'. In view of the abovementioned results, this is due to contralateral dRGCs.

### dRGCs response to injury

Using two different models of RGC injury, one direct trauma to the optic nerve and the other increase of the intraocular pressure, we show here that dRGCs die within the same temporal and spatial pattern than oRGCs. Thus, dRGCs are not more resilient to injury than oRGCs.

### Topography

The ipsilateral projection is involved in binocular fusion. Because in rats and mice the binocular field is situated above the head, the binocular area of the retina is ventro-temporal, with the shape of a moon crescent (Lund, 1965; Dräger and Olsen, 1980; Nadal-Nicolás et al., 2012). In the pigmented strain, the crescent is denser and covers a wider retinal area, reaching almost to the optic nerve (see Figures 6I,J,K,M). Ipsilateral dRGCs are also found in this retinal area. There are as well, aberrant RGCs, both dRGCs and oRGCs, which are those traced from the ipsilateral SC that do not fall within the retinal crescent (Dräger and Olsen, 1980; Balkema and Dräger, 1990).

With respect to all dRGCs, Dräger and Olsen (1980) showed that in pigmented and albino mice they are mostly peripheral and ventral, but in their Figures 11, 12 it is observed that there is a peak of dRGCs in the temporal retina of pigmented mice, a peak that is not so obvious in the albino.

**Figure 11:**
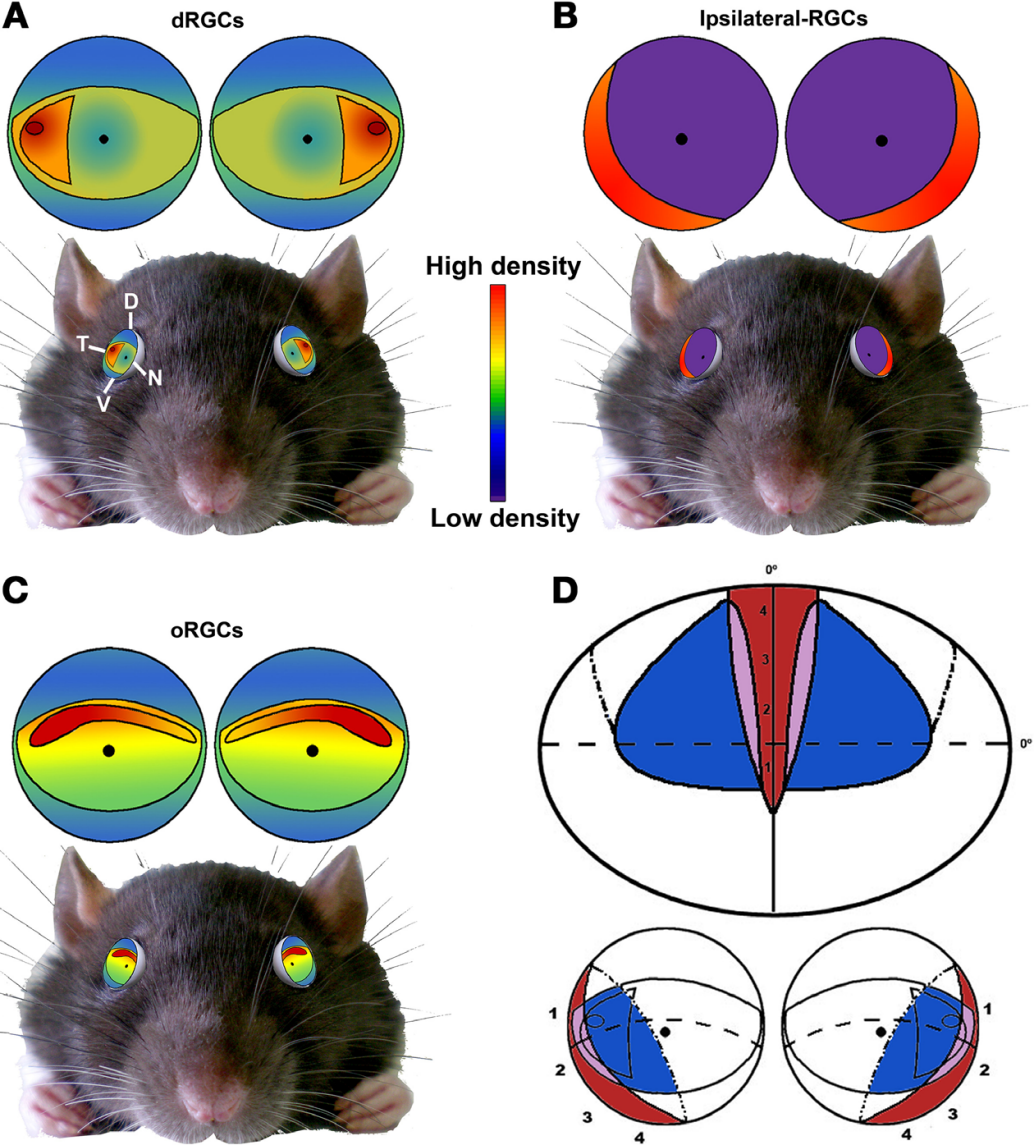
**Summary**. Schematic drawings showing the distribution of dRGCs **(A)**, ipsilateral RGCs **(B)** and oRGCs **(C)** and their position in the rat eye (bottom). **(D)** Scheme of the rat visual field (top) and its representation in the retina (bottom). Red, ipsilateral field; blue, dRGCs field; purple, both. Adapted from Coleman et al. (2009) and Dräger and Olsen (1980) D, dorsal; V, ventral; T, temporal; N, nasal.

Here we show that in both rat strains ~50% of the dRGCs lay in the temporal retina. This is in contradiction to previous works where it was reported that rat dRGCs are encountered across the retina but more frequently toward the periphery (Liu and Jen, 1986; Buhl and Dann, 1988). Liu and Jen analyzed albino animals, and in this strain the temporal area is less visible than in pigmented maps, although clearly visualized in the normalized maps and resulting graphs (Figures 4I,J, 5). Buhl and Dann worked with a pigmented strain, but they did not report total numbers or a detailed distribution since their purpose was to determine the anatomical attributes of dRGCs. Our maps show that dRGC distribution is very precise, they are found in the retinal central equator (Figures 4, 5–7, 11A) and within it, there is a vertical ellipse in the temporal retina of higher dRGC density. In some maps, it seems that the elliptical high density area is either ventral or dorsal, but this may be due to the cuts made in the retina to flat-mount it. A small deviation a little to the right or to the left from the exact medial position would shift it.

Interestingly, the highest density of dRGCs is placed in the area of highest oRGC density: the temporal tip of the visual streak (Nadal-Nicolás et al., 2009, 2012; Salinas-Navarro et al., 2009c; Ortín-Martínez et al., 2010), a little above of the optic nerve.

### Function

In birds, reptiles and amphibians, dRGCs are responsible for the optokinetic nystagmus and they always project to the accessory optic nuclei (Simpson, 1984; Cook and Podugolnikova, 2001). In mammals, their function is unknown. It is believed that they are displaced by an ontogenic mistake. This is based in two facts: i/ dRGCs are a highly diverse population, anatomically resembling most of the oRGC subtypes (Dräger and Olsen, 1980; Liu and Jen, 1986; Buhl and Dann, 1988; Balkema and Dräger, 1990; Doi et al., 1995), and, ii/ the dendritic coverage of the whole retina, necessary for a group of RGCs to be considered a functional subtype, is 1.1 for rat dRGCs (Buhl and Dann, 1988).

Because rat dRGCs are a heterogeneous group of RGCs and their dendritic coverage is one-fold, Buhl and Dann (1988) concluded that this excluded the possibility of processing spatially independent information for any given type of dRGC. However, they calculated the dendritic coverage based on an homogeneous dRGC distribution.

A recent report (Bleckert et al., 2014) shows that in mice alpha-like RGCs have a nasal to temporal density gradient that facilitates visual sampling in the frontal view, and conclude that in mice RGCs topographies are designed to scan the space differently and thus increasing the spatial sampling of the binocular visual field. So, a given RGC type does not have to be represented in the whole retina to perform its function.

Based on our neighbor maps (number of cells in a radius of 0.276 mm, see Materials and Methods), in the equatorial retina area occupied by dRGCs, their minimum density is 4 dRGCs/mm^2^ in albino rats and 58 dRGCs/mm^2^ in pigmented. In the temporal region, the maximum density is 30 and 245 dRGCs/mm^2^, respectively. Assuming the averaged dendritic field of dRGCs published by Buhl and Dann (0.045 mm^2^) (Buhl and Dann, 1988), then in albino rats their dendritic coverage will range from 0.2 to 1.3, and in pigmented rats from 2.6 to 11. Thus, in pigmented rats in the temporal retina, the dendritic coverage of dRGCs is very high, and therefore different dRGCs subtypes might be functionally independent.

Recently, Wallace et al. (2013) discovered that rats move constantly and asymmetrically their eyes to keep the visual field above them continuously overlapping but not continuously aligned, as primates or animals with frontal eyes do. This behavior would explain the relatively poor ipsilateral projection in rats. Evolutionary this benefits the animal, since having at all times a clear overhead view is essential for predator detection.

In Figure 11 is shown a summary of the distribution of dRGCs, ipsilateral RGCs and oRGCs in the rat, their position in the eye and the rat binocular visual field. oRGCs are more abundant in a horizontal visual streak that runs naso-temporally in the dorsal retina. Presumably this horizontal area would provide a clear frontal vision and might be used in tasks that need forward vision such as gap jumping (Wallace et al., 2013). On the other hand, dRGCs are mainly located in the temporal equator, and when the eye torsions to gaze overhead, they will be in the perfect position to scan the sky. The faster a predator is detected the better, so even a minute difference in the speed of response is important.

Having all this in mind, we propose that dRGCs are essential to integrate the overhead visual information. Because albino animals are visually impaired and their reduction in RGCs is mostly due to a diminished dRGC population, one way to support our hypothesis would be to compare the speed and accuracy of albino vs. pigmented rats in detecting predators coming from above.

## Author contributions

All authors have reviewed and approved the final version of this work. Conceptualized and designed the experiments: Francisco M. Nadal-Nicolás; Manuel Vidal-Sanz; María P. Villegas-Pérez, Marta Agudo-Barriuso. Performed the experiments: Francisco M. Nadal-Nicolás, Paloma Sobrado-Calvo, Manuel Salinas-Navarro, Manuel Jiménez-López. Data acquisition: Francisco M. Nadal-Nicolás, Paloma Sobrado-Calvo. Data analysis: Francisco M. Nadal-Nicolás, Manuel Salinas-Navarro, Marta Agudo-Barriuso. Design of automated routines: Manuel Jiménez-López. Data interpretation, manuscript drafting: Francisco M. Nadal-Nicolás, María P. Villegas-Pérez, Manuel Vidal-Sanz, Marta Agudo-Barriuso. Contributed reagents/materials/analysis tools: María P. Villegas-Pérez, Manuel Vidal-Sanz, Marta Agudo-Barriuso.

### Conflict of interest statement

The authors declare that the research was conducted in the absence of any commercial or financial relationships that could be construed as a potential conflict of interest.

## Supplementary material

Supplementary Figure 1 **Confocal images from an albino (A) and a pigmented (B) flat mounted retina showing FG-traced (white) and Brn3a^+^ (red) oRGCs (square)**. In the *z* plane (right and bottom, rectangular images) dRGCs are observed. These images were taken using a Nikon Eclipse 90i confocal microscope (Servicio de Apoyo a la Investigación, Universidad de Murcia, Spain).
