# Supplementary figures and images for: Displaced retinal ganglion cells in albino and pigmented rats
Source: Front Neuroanat. 2014 Oct 6;8:99. doi: 10.3389/fnana.2014.00099 (PMC4186482; doi:10.3389/fnana.2014.00099)

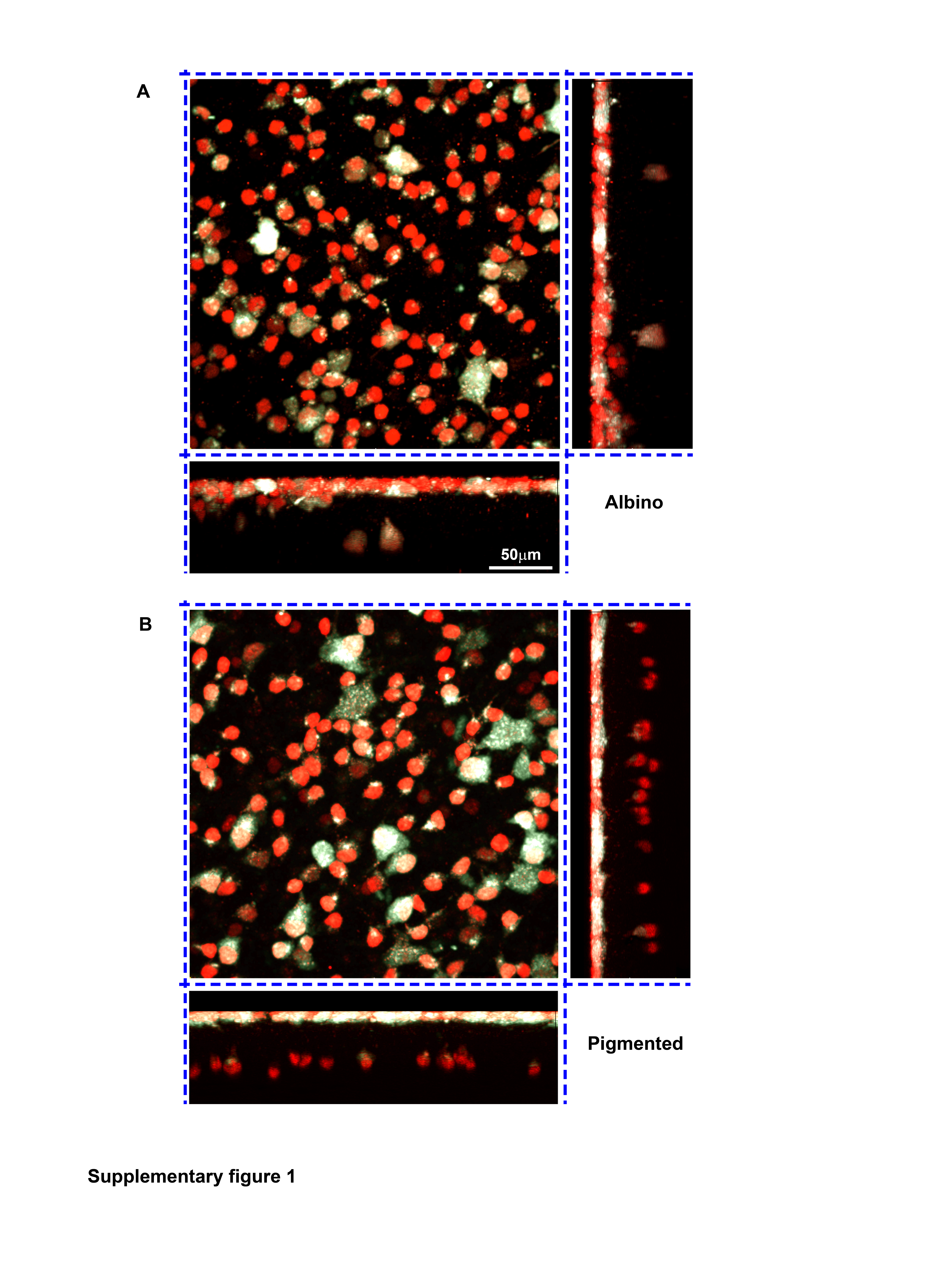

Supplement: Supplementary Figure 1 — Confocal images from an albino (A) and a pigmented (B) flat mounted retina showing FG-traced (white) and Brn3a+ (red) oRGCs (square). In the z plane (right and bottom, rectangular images) dRGCs are observed. These images were taken using a Nikon Eclipse 90i confocal microscope (Servicio de Apoyo a la Investigación, Universidad de Murcia, Spain). [file Image1.JPEG]
